# Dynamic Control of DNA Origami Self-Assembly by Transcriptional Modules

**DOI:** 10.1021/jacs.5c18964

**Published:** 2026-01-07

**Authors:** Lei Zhang, Ruojie Sha, Lev Bershadsky, Paul M. Chaikin

**Affiliations:** † Department of Physics, 5894 New York University, New York, New York 100038, United States; ‡ Department of Chemistry, New York University, New York, New York 100038, United States

## Abstract

Biological cells achieve adaptive and responsive behaviors by dynamically regulating self-assembly through sensing, processing, and transmitting environmental information. Emulating this is key to engineering dynamic synthetic materials with life-like functions. In most existing dynamic self-assembly systems, the responses are achieved by changes in the free energy landscape induced by external inputs (such as molecules, light, or pH) that push the system toward a new stable thermodynamic equilibrium. In contrast, achieving the sustained and complex processes characteristic of living systems requires a nonequilibrium approach involving continuous energy dissipation. Here, we present a new strategy for dynamic control of DNA origami tile self-assembly by directly coupling a transcriptional module’s activity to the tiles’ assembly state. Transcription is triggered only upon tile dimerization, which brings the module components into close proximity. The resulting RNA blocker strands then disassemble into dimers via strand displacement, establishing a dissipative, autonomous feedback loop. We demonstrated that integrating two mutually inhibitory tile pairs constructs a bistable system whose state can be switched by using RNA inducers or upstream transcriptional circuits. Simulations of larger networks further predict complex, nonequilibrium temporal behaviors (including sustained oscillations and pulses) that are maintained only through continuous energy consumption. This work presents a generalizable strategy for dynamic control of DNA origami tile self-assembly via transcriptional modules, paving the way for applications in nanorobotics, biosensing, biomedicine, and artificial life systems.

## Graphical abstract

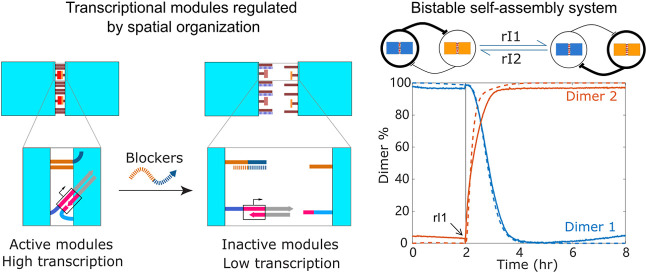

## Introduction

Besides carrying genetic information through its double-stranded duplexes, the programmability of DNA base pairing opened the field of DNA nanotechnology,^1^ helping the development of programmable self-assembling systems.^2^ Various DNA nanostructures, such as DNA crystals,^3,4^ DNA bricks,^5^ DNA nanotubes,^6,7^ and DNA origami,^8,9^ have been studied to organize in programmable mode, allowing the construction of molecular objects with controlled nanoscale features. Especially, DNA origami enables highly precise and programmable self-assembly of complex nanostructures,^10^ making it ideal for applications in targeted drug delivery,^11,12^ biosensing,^13,14^ nanorobotics,^15,16^ and computation.^17,18^

In addition to responsive DNA assemblies that operate via DNA strand hybridization and exchange triggered by input strands or other external stimuli^19^—transitioning from high- to low-energy states under equilibrium thermodynamics,^20^ which limits them to passive state changes—nonequilibrium, energy-consuming assemblies have also been developed.^21^ Like living systems,^22,23^ which function far-from-equilibrium by continuously consuming energy through cycles of assembly and disassembly,^24^ this dissipative approach enables systems to adapt and respond dynamically to their environment and perform sustained work.^25^ Dissipative self-assembly, fueled by chemical energy,^21,26^ enables transient, reversible, and autonomous behaviors, mimicking life-like functions such as motion,^27,28^ adaption,^29^ and dynamic reconfiguration.^30,31^

In vitro transcriptional circuits built from synthetic transcriptional templates (genelets)^32^ are powerful tools for achieving complex temporal dynamics such as bistability,^32–34^ pulses,^35,36^ and oscillations.^31,37,38^ Transcription of genelets is available only when activated by specific DNA strands, and the resulting RNA transcripts regulate other genelets. RNase-mediated degradation ensures dynamic turnover, mimicking natural gene network regulation. These RNA signals have been studied to control downstream materials, including dynamic DNA nanotubes assemblies^38,39^ or reversible release of gold nanoparticles from 3D DNA frameworks.^40^ In these previous examples, the transcriptional circuits acted as an external controller where the RNA signals from these circuits drive the downstream responses. Despite these achievements, another approach—where the structure itself provides the regulatory signal to control DNA origami self-assembly—remains largely underexplored.

Here, we demonstrate the dynamic control of DNA origami tile self-assembly using transcriptional modules whose activity is regulated by spatial organization. By directly integrating these modules into the DNA origami tiles’ self-assembly process, we establish a feedback loop where the modules’ activity is regulated by the tiles’ assembly state. DNA origami tiles^41^ in this work were represented by squares (Figure 1a). Each transcriptional module consists of a genelet with an incomplete T7 RNA polymerase (T7 RNAP) promoter and a short DNA activator strand. Direct integration of the transcriptional modules into the DNA origami tile self-assembly process enables dynamic control of the overall self-assembly. Unlike most previous transcriptional circuits^31–38^ where the genelet’s transcription activity is regulated by a long DNA activator strand (typically 25 bases) that can bind stably to complete the promoter site for T7 RNAP (Figure 1b), the DNA activator strand in our transcriptional modules is significantly shorter (6 bases). Consequently, this short activator cannot stably bind to the gene to complete the promoter site and initiate transcription (Figure 1c). Transcription activity of this module can be coupled to tile self-assembly by tethering it to separated DNA origami tiles that dimerize via hybridization of 4 sticky ends: tile dimerization brings the genelet and activator into sufficient proximity, increasing the local concentration and facilitating their stable binding to enable transcription (Figure 1d). To achieve reversible and tunable control, toehold domains were appended to the sticky ends. Blocker strands can invade sticky ends by hybridization by a toehold-mediated strand displacement reaction (Figure 1e), thereby dissociating the dimer and suppressing transcription. Furthermore, we sought to build DNA origami tiles assemblies with autonomous feedback regulation. The blocker strands can be produced by transcriptional modules integrated on the same dimer, enabling the transcribed blocker strands to regulate the assembly process itself (Figure 1f). Finally, we combined two tile pairs whose transcripts inhibit each other (Figure 1g), creating a dynamic bistable self-assembly system whose state can be switched by either RNA inducers or external transcriptional circuits.^35^ This work introduces a new design paradigm for responsive nucleic acid materials by integrating control modules with the physical state changes of DNA nanostructures.

**Figure 1:**
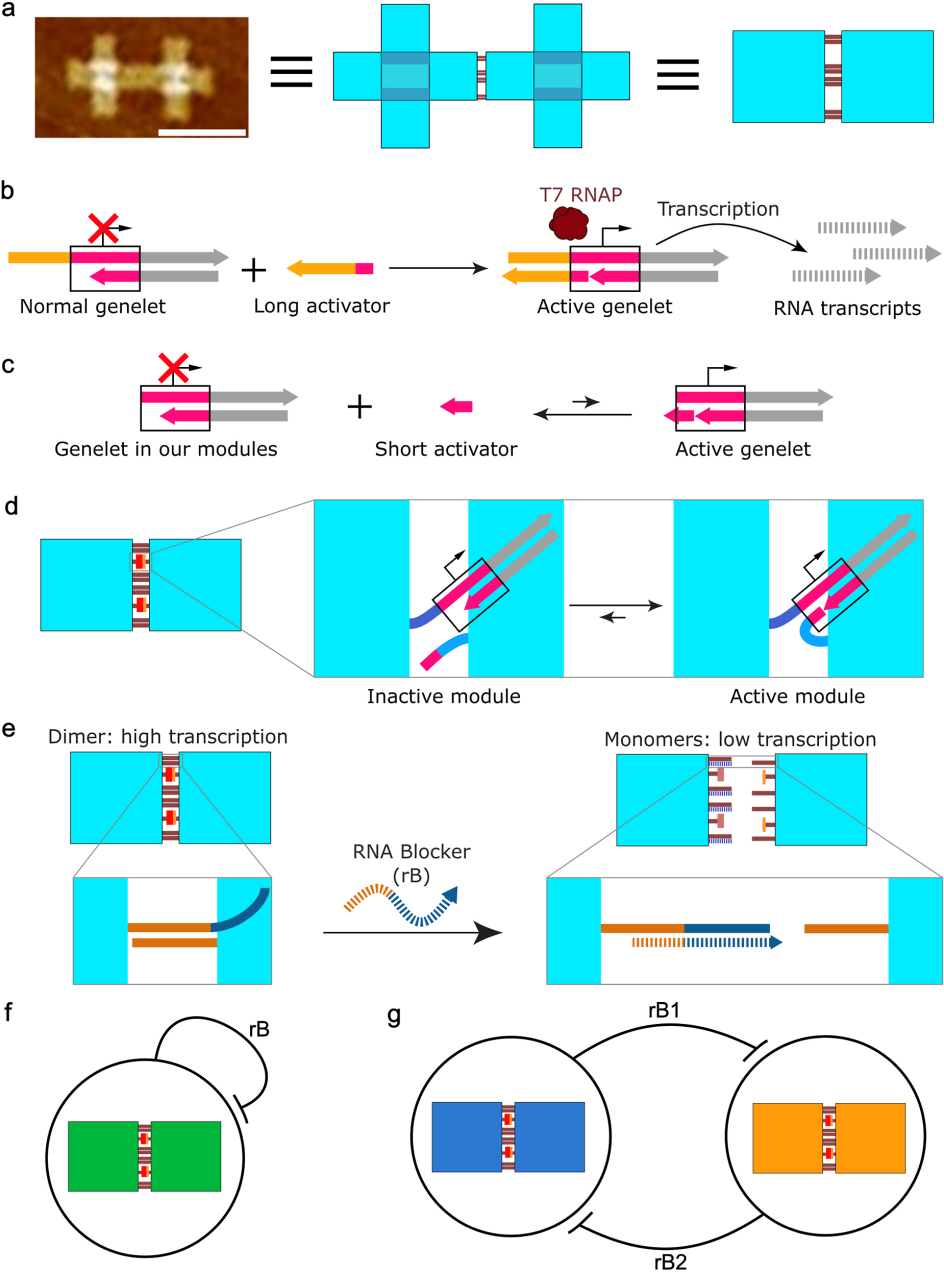
Design of dynamic control of DNA origami tiles self-assembly by transcriptional modules. (a) DNA origami tile dimer in this work. DNA origami tiles are represented by squares unless otherwise stated. Scale bar, 100 nm. (b) The genelet is a synthetic transcriptional template containing an incomplete promoter site for T7 RNA Polymerase (T7 RNAP). In most previous transcriptional circuits, the genelet’s transcription activity is regulated by a long DNA activator strand. This activator binds stably to the genelet, thereby completing the promoter site and enabling transcription. (c) In our transcriptional modules, the DNA activator strand is significantly shorter (6 bases). Consequently, it cannot bind stably to the genelet to complete the promoter site. (d) Transcriptional modules whose activities are coupled with DNA origami tiles self-assembly. Each transcriptional module consists of a genelet and a short DNA activator strand, each tethered to separated DNA tiles. Tiles dimerization brings the genelet and activator into close proximity, increases the local concentration, and enables stable binding for transcription. (e) When blocker strands dissociate the dimer by interfering with the sticky ends, the transcription is inhibited. (f) Tiles self-assembly with feedback-regulation. The blocker strands (rB) can be produced by integrated transcriptional modules on the same dimer, enabling feedback-regulated self-assembly. (g) A dynamic bistable tiles self-assembly system can be constructed by coupling two tile pairs whose transcripts (rB1 and rB2) dissociate each other.

## Results and Discussion

### DNA Origami Tiles Can Keep Integrity with T7 RNAP

T7 RNAP has been reported to bind and transcribe a wide range of DNA sequences nonspecifically,^42^ which can lead to undesired disassembly or aggregation of DNA nanostructures.^43,44^ Therefore, we first checked whether DNA tiles can maintain their structural integrity and function in the presence of T7 RNAP. We analyzed the DNA origami structures by using agarose gel electrophoresis. When a mixture of DNA origami dimers and monomers was incubated with T7 RNAP, some nonspecific transcribed RNA can serve as linkers, leading to clustering of the tiles (Figure S2a, Lane 3). Upon the addition of RNase H, which degrades unwanted RNA linkers, the original band pattern was restored (Figure S2a, Lanes 4 and 5), indicating that the tiles maintained their structural integrity and that nonspecific RNA-induced clustering could be effectively reversed. AFM imaging further confirmed that both DNA origami monomers and dimers preserved their shape after more than 20 h of incubation with T7 RNAP and RNase H (Figure S2b,d).

### Design Transcriptional Modules Regulated by Spatial Organization

We next demonstrated that highly efficient transcription occurs only upon dimer formation (Figure 2a). To test this, we compared transcription rates between two conditions: tiles with complementary sticky ends that form dimers and tiles without complementary sticky ends that remain as monomers. For real-time monitoring of RNA production, we developed a fluorescent DNA duplex reporter, in which the transcribed RNA displaces a quencher-labeled DNA strand, leading to a significant fluorescence increase (Figure 2b). An effective transcription module should exhibit high transcription in the dimer state and minimal leaking transcription in the monomer state. A key factor influencing the transcription efficiency is the length of the activator strand. There is a trade-off: if the strand is too short, it cannot stably bind to the genelet even in dimers, resulting in low transcription; if too long, it may bind the genelet in monomers, leading to high unwanted leaking transcription. We tested activator strands of 5, 6, and 7 bases (Figure 2c) and selected the 6-base design for subsequent experiments, as it provided the best balance between high transcription in dimer and low leaking transcription in monomers (Figure 2d).

**Figure 2:**
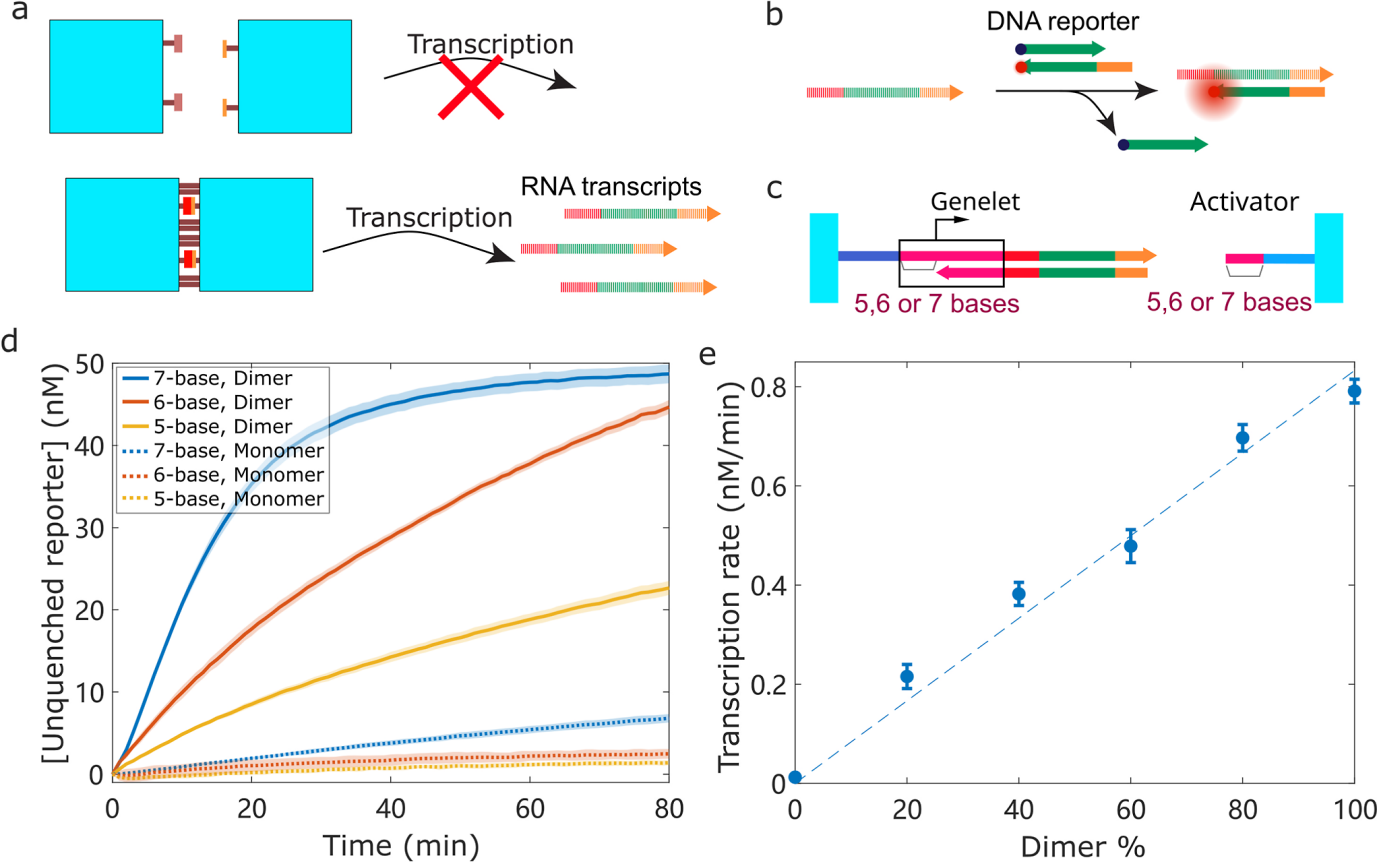
Transcriptional modules’ activity can be regulated by dimerization. (a) Highly efficient transcription by transcriptional modules is available only upon dimerization of DNA origami tiles by complementary sticky ends. (b) Quantitative measurement of transcriptional rate using a reporter complex (dark blue dot, quencher; orange dot, fluorophore). (c) Transcriptional modules with different numbers of lacking bases (5, 6, and 7 bases) in the promoter domain and corresponding activator strands. (d) Activity of transcription modules of 5-, 6-, and 7-base design in dimer and monomer state were measured, respectively. 6-Base design provided the best balance between high transcription in the dimer state and low leaking transcription in the monomer state. Solid and dotted curves are the averages of 3 measurements. Shaded regions represent standard variations of 3 measurements. (e) RNA production rates with different dimer percentages. Error bars represent standard variations of 3 measurements. The dashed line is a linear regression model used to validate that RNA production rates are proportional to the percentages of tiles in the dimer state (*R* ^2^ = 0.9811).

The transcription rate should be proportional to the fraction of dimers, as this fraction determines the concentration of active genelets capable of producing RNA. We mixed 5 nM tiles containing genelets with varying amounts (0, 1, 2, 3, 4, or 5 nM) of tiles carrying the activator strand, thereby generating a series of samples with increasing dimer percentages. After adding T7 RNAP, we measured the RNA production rates and found that they increased proportionally with the dimer percentages, i.e., with the concentration of active genelets (Figures S4 and 2e).

### Reversible Tiles Dimerization Directed by Programmable Nucleic Acid Reactions

We next aimed to regulate dimer dissociation using blocker strands. As shown in Figure 1e, blocker strands dissociate dimers by invading sticky-end hybridization through a toehold-mediated strand displacement reaction. To examine the concentration dependence of these blockers, we added varying concentrations of the DNA blocker (dB) strands to 5 nM dimers and quantified the percentage of dimers using agarose gel electrophoresis (Figure 3a). Progressive dimer disassembly was observed as the blocker strand concentration increased. To better understand this behavior, we developed a quantitative model to describe the relationship between the blocker strand concentration and dimer dissociation. See Supporting Information Section 12.2 for more information about this model.

**Figure 3:**
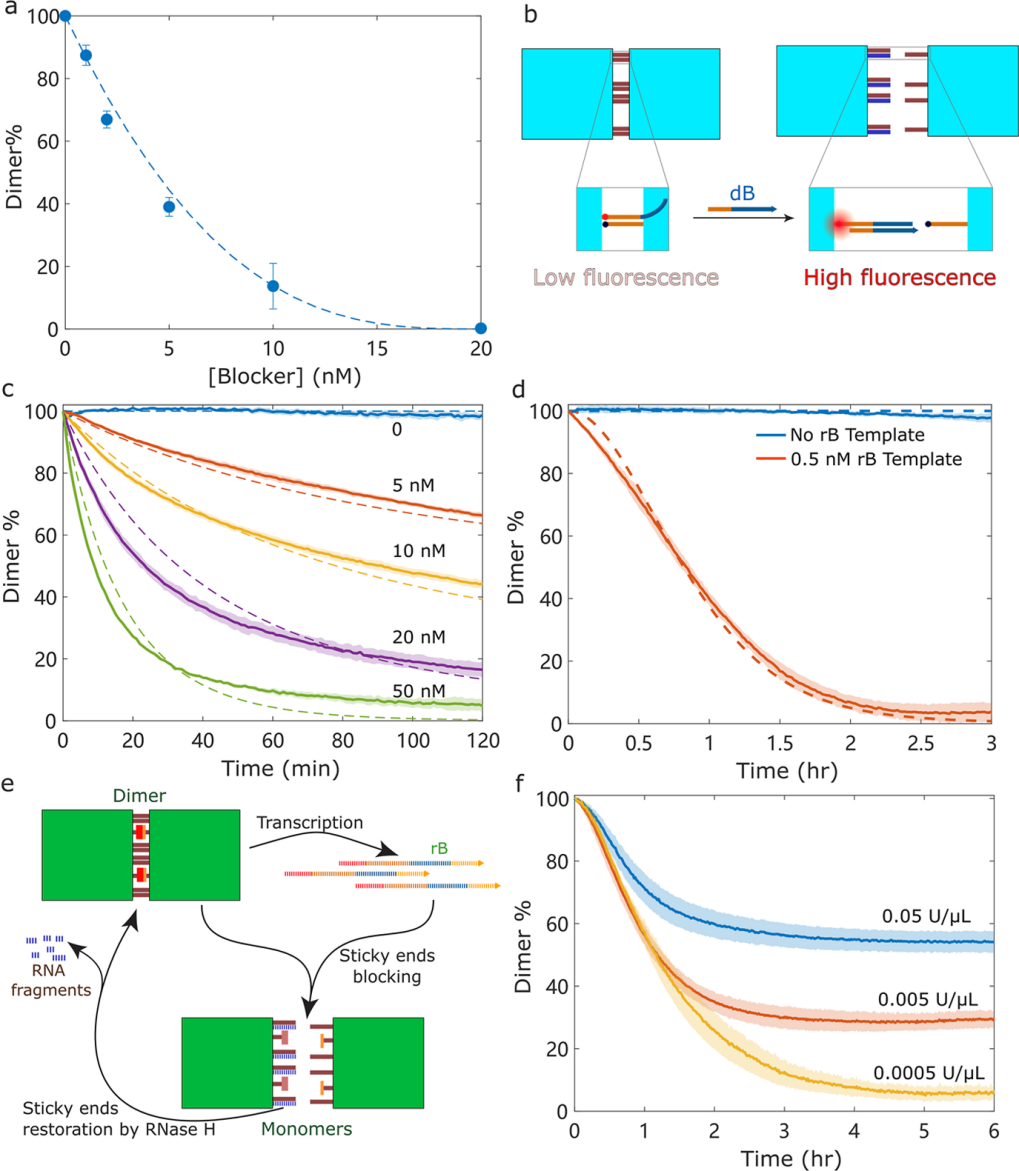
Reversible dynamic control of DNA origami tiles assembly. (a) Increasing the concentration of blocker strands led to progressive disassembly of the dimers. Error bars represent standard variations of 4 measurements. (b) A fluorophore and a quencher are incorporated into the complementary sticky ends of two tiles to monitor the dimerization by which fluorescence is quenched by the closing quencher. (c) Kinetics of blocker-directed dimer dissociation across different blocker concentrations. (d) Kinetics of in vitro transcription directed dimer dissociation. (e) Tiles assembly with feedback regulation. RNA transcripts produced by one dimer promote its own dissociation. Meanwhile, RNA blockers are degraded by RNase H, which restores the tiles’ sticky ends. (f) By adjusting the RNA degradation rate through varying RNase H concentrations, both the dissociation dynamics and steady-state levels can be tuned. Solid lines in Figure 3c,d and f represent experimental results. Dashed lines in Figure 3a,c and d represent simulations. Shaded regions in Figure 3c represent two individual replicates. Shaded regions in Figure 3d and f represent standard variations of 3 measurements.

In addition to studying equilibrium dissociation levels, we also investigated the kinetics of dimer formation and dissociation. To monitor these dynamics in real time, we incorporated a fluorophore and a quencher into the complementary sticky ends of two tiles (Figure 3b). When the tiles are dimerized, the fluorophore and quencher are close, leading to fluorescence quenching. When the dimers are dissociated due to the addition of blocker strands, the fluorophore and quencher are far apart, resulting in a high fluorescence intensity. To validate that the fluorescence signal accurately reflects dimerization levels, we compared fluorescence data with results from agarose gel electrophoresis (Figure S5). Samples with known ratios of dimers were split for parallel analysis by the two methods, both of which yielded consistent measurements of dimer percentages (Figure S6). We then used fluorescence to track the kinetics of dimer formation (Figure S18b) and blocker-induced dimer dissociation across different blocker concentrations (Figure 3c) and developed a model to describe the kinetics of dimerization and dissociation. See Supporting Information Section 12.3 for more information about this model.

### Dimer Dissociation Regulated by In Vitro Transcription

As the next step in dynamic control of DNA origami tiles self-assembly, we investigated whether blocker strands could be RNA (rB) transcribed in situ (Figure S7). When T7 RNAP was added to the dimers alone, the fluorescence signal remained unchanged, indicating no dissociation. However, upon addition of 0.5 nM rB Template, a significant increase in fluorescence was observed, confirming RNA-triggered dimer dissociation (Figure 3d).

To introduce feedback control, we next coupled dimerization-regulated transcription with transcription-regulated dimerization—such that RNA transcripts produced from one dimer promote its own dissociation (Figure 3e). Meanwhile, blocked sticky ends can be restored when the hybridized RNA blocker is degraded by RNase H. As dimers dissociate, the RNA blocker production rate decreases because the transcriptional modules are suppressed in the monomer state; however, its degradation rate increases due to more RNA blocker/DNA sticky ends hybrid as a substrate for RNase H. The system approaches a steady state when these two rates are balanced. By adjusting the RNA degradation rate through varying RNase H concentrations, both the dissociation dynamics and the steady-state levels can be tuned: a higher RNase H concentration leads to faster RNA blocker degradation, which in turn slows the dimer dissociation rate, allowing the system to approach a steady state with a higher remaining dimer percentage (Figure 3f).

### Construct a Dynamic Bistable Tiles Self-Assembly System

Combining multiple tile pairs that regulate each other’s assembly and transcription activity enables the construction of systems with diverse dynamic behaviors.^35^ As a demonstration, we designed a dynamic bistable system by combining two tile pairs whose RNA transcripts mutually trigger each other’s dissociation (Figure 4a). Transcribed RNAs from one dimer serve as RNA blockers (rB) to another dimer to trigger its dissociation by blocking its sticky ends and to inhibit the corresponding transcriptional modules. In addition to signal production via transcription, RNA signals are also degraded by RNase H for signal turnover. By choosing correct conditions (tile concentrations and transcription and degradation rates), such a system can exhibit bistability; i.e., this system will be attracted to one of the two stable steady states where only one tile pair is predominantly dimerized while the other is dissociated, resulting in high expression from the corresponding transcription modules. We defined State 1 (S1) as the state characterized by the prevalence of dimerized tile pair 1 and monomeric tile pair 2 and State 2 (S2) as the state characterized by the prevalence of dimerized tile pair 2 and monomeric tile pair 1.

**Figure 4:**
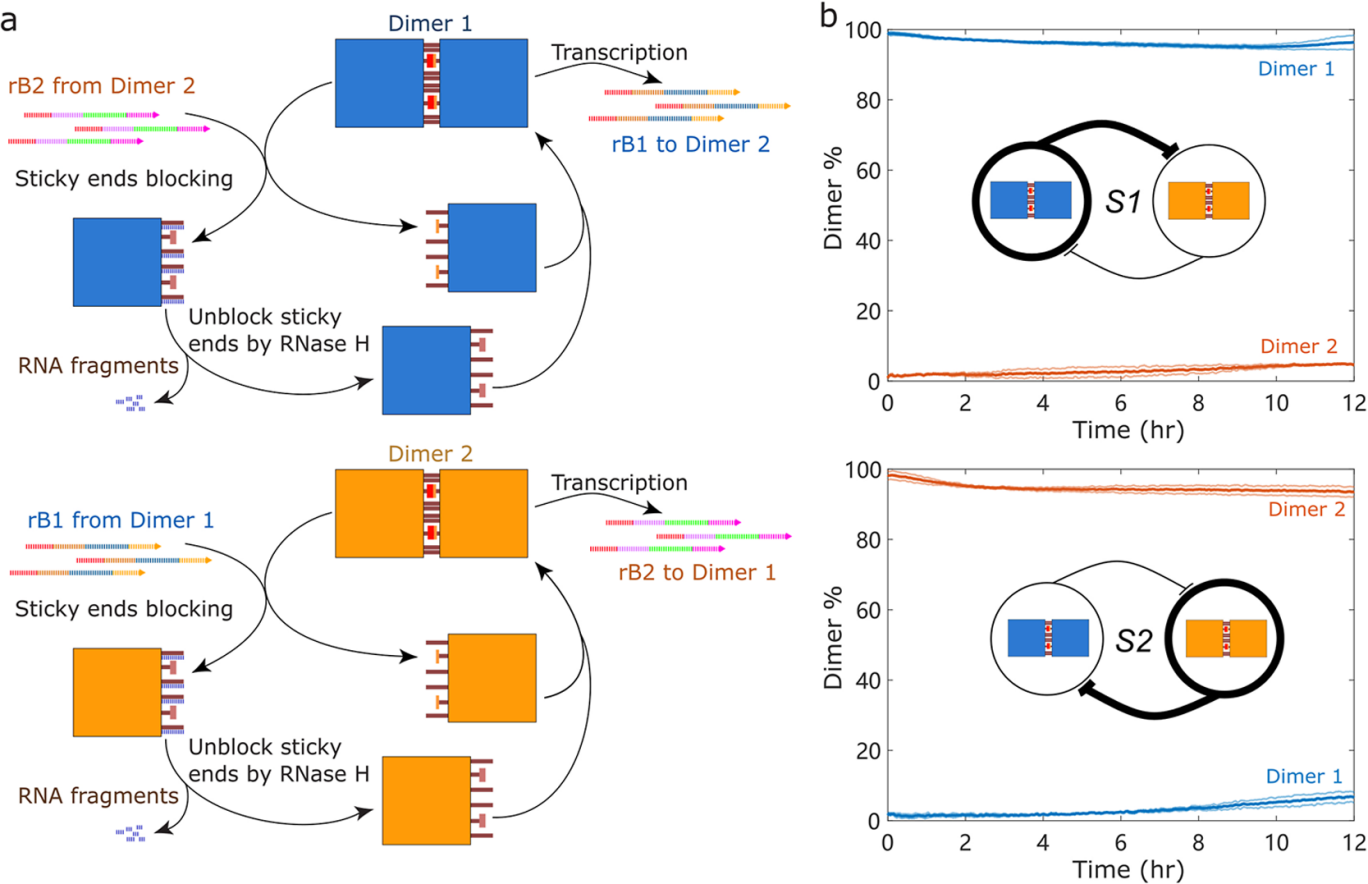
Design of a bistable DNA origami tiles self-assembly system. (a) Two tile pairs with transcriptional modules were combined whose RNA transcripts mutually trigger each other’s dissociation. Transcribed RNAs from one dimer serve as the RNA blockers to another dimer to trigger its dissociation by blocking its sticky ends and to inhibit the corresponding transcriptional modules. In addition to signal production via transcription, RNA signals are also degraded by RNase H for the signal turnover. (b) Distinct states (S1 or S2) can be achieved by adding the corresponding RNA blockers at the start, and the resulting state is maintained for at least 12 h. Solid lines represent the average of two measurements; thin lines represent two individual replicates. Bold blunt arrows indicate the dominant dimer and high RNA expression.

To track the Dimer % of each tile pair, tile pair 1 and tile pair 2 were incorporated with different fluorophore/quencher pairs: Cy3/BHQ_2 and Cy5/IAbRQ, respectively. See Supporting Information Section 8 for details of the bistable system quantification. Distinct states (S1 or S2) can be achieved by adding the corresponding RNA blockers (rI1 or rI2) at the start, and the resulting steady state is maintained for at least 12 h (Figure 4b).

### Self-Assembly State Can Be Switched by RNA Inducers

We further explored whether this bistable system could switch states in response to external stimuli, just like cells switch between different gene expression programs in response to environmental stimuli.^45,46^ As a first step, we manually added inducer RNA strands (rI), which are designed to repress the action of the current expressed blockers. If the current system is in S1, rI1 can inhibit free RNA blockers (rB1) in solution through nucleic acid hybridization and displace rB1 from Dimer 2’s sticky ends (Figures 5a and S8), thereby promoting Dimer 2 formation. The rB2 transcribed by Dimer 2 subsequently dissociates Dimer 1 into monomers, switching the system into S2.

**Figure 5:**
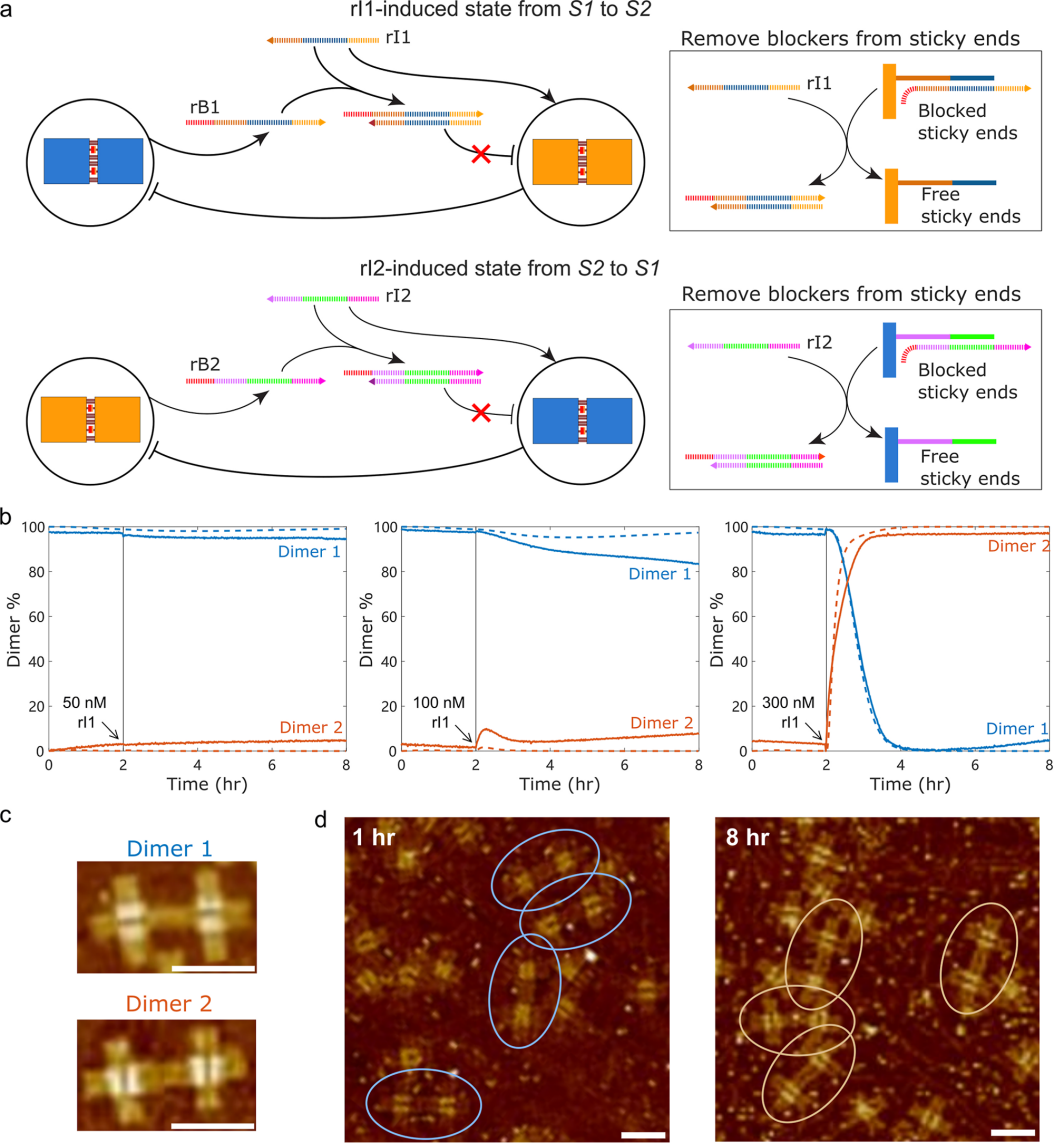
State switching by RNA inducers. (a) Self-assembly state can be switched by adding inducer RNA strands (rI) that inhibit the free blocker strands (rB) in solution through nucleic acid hybridization and displace rB from sticky ends on another tiles pair. (b) The concentration of inducers has a significant influence on the bistable dynamics. 300 nM rI1 can switch the state from S1 to S2 after incubation for 2 h, but 50 and 100 nM rI1 failed. Solid lines represent experimental results; dashed lines represent simulations. (c) Identification of two distinct dimers under AFM. Dimer 1 is characterized by parallel horizontal equal signs (“ = = ”), while Dimer 2 shows parallel vertical equal signs (“|| ||”). Scale bar, 100 nm. (d) AFM images acquired at 1 h (S1) and 8 h (S2) confirm the transition from S1 (Dimer 1 dominant) to S2 (Dimer 2 dominant) during the state switching process in Figure 5b, right panel. Scale bar, 100 nm.

The concentration of inducers has a significant influence on the switching dynamics (Figure 5b). The system is set as S1 when initially adding rB1. After adding T7 RNAP and incubating for 2 h, 300 nM rI1 successfully induced the state from S1 to S2. In contrast, lower concentrations (e.g., 50 nM and 100 nM) were insufficient to trigger the state switch. The state switch from S2 to S1 is also available (Figure S14).

The required amount of RNA inducers also depends on the incubation time following the addition of T7 RNAP (Figure S15). For example, while 300 nM rI1 could effectively switch the state from S1 to S2 after a 2 h incubation, the same concentration failed to switch the state after 5 h due to the accumulation of more rB1 over time. In this case, 500 nM rI1 is required to induce state switching. To account for the dependence of inducer RNA concentrations and incubation time when switching such a bistable system, we developed a kinetic model combining reversible dimerization and transcriptional modules’ activity based on ordinary differential equations (ODEs) describing the mass-action kinetics of dimer formation and dissociation, RNA transcription and degradation, and RNA-induced strand displacement. This model can accurately capture the dynamics of state switch in response to different concentrations of inducer provided at different times. See Supporting Information Section 12.5 for details of this model.

We further characterized the actual structures of the tiles self-assembly system during state switching by AFM imaging. Dimer 1 and Dimer 2 could be distinguished by their equal signs layouts: Dimer 1 was identified by its parallel horizontal motif (“ = = ”), while Dimer 2 identified by its parallel vertical motif (“|| ||”) (Figure 5c). To confirm the switching process, we repeated the state switching as the right panel of Figure 5b by adding 300 nM rI1 at 2 h and took AFM images of the sample acquired at 1 h (S1) and 8 h (S2), respectively (Figure 5d). These results confirmed a predominant population of Dimer 1 in S1, followed by a shift to the dominance of Dimer 2 in S2.

### Self-Assembly State Can Be Switched by Upstream Transcriptional Networks

We next aimed to switch system states using additional transcriptional networks, enabling integration with other in vitro transcriptional circuits to achieve more complex dynamics and functionality.^47^ Specifically, we designed transcriptional modules that produce inducer RNA strands (Figure S16) based on previous work by Schaffter et al.^35^ These modules are initially inactive, but their transcription can be selectively activated or inhibited by the addition of activator or repressor strands—allowing this system to be embedded within larger dynamic transcriptional networks. The system was stable in both initial states and could be reliably switched in either direction by activating the corresponding Inducer Genelets (Figure 6a,b). The new state was maintained even after the Inducer Genelets was turned off. Moreover, the system could be switched multiple times by sequentially activating and deactivating the relevant Inducer Genelets (Figure 6c). We further performed AFM imaging on samples acquired at 1, 4.5, and 10 h throughout this state switching process (Figure 6d). These results verified a transition from the initial state S1 (Dimer 1 with “ = = ” motif takes dominance) to S2 (Dimer 2 with “|| ||” motif takes dominance) and finally back to S1.

**Figure 6:**
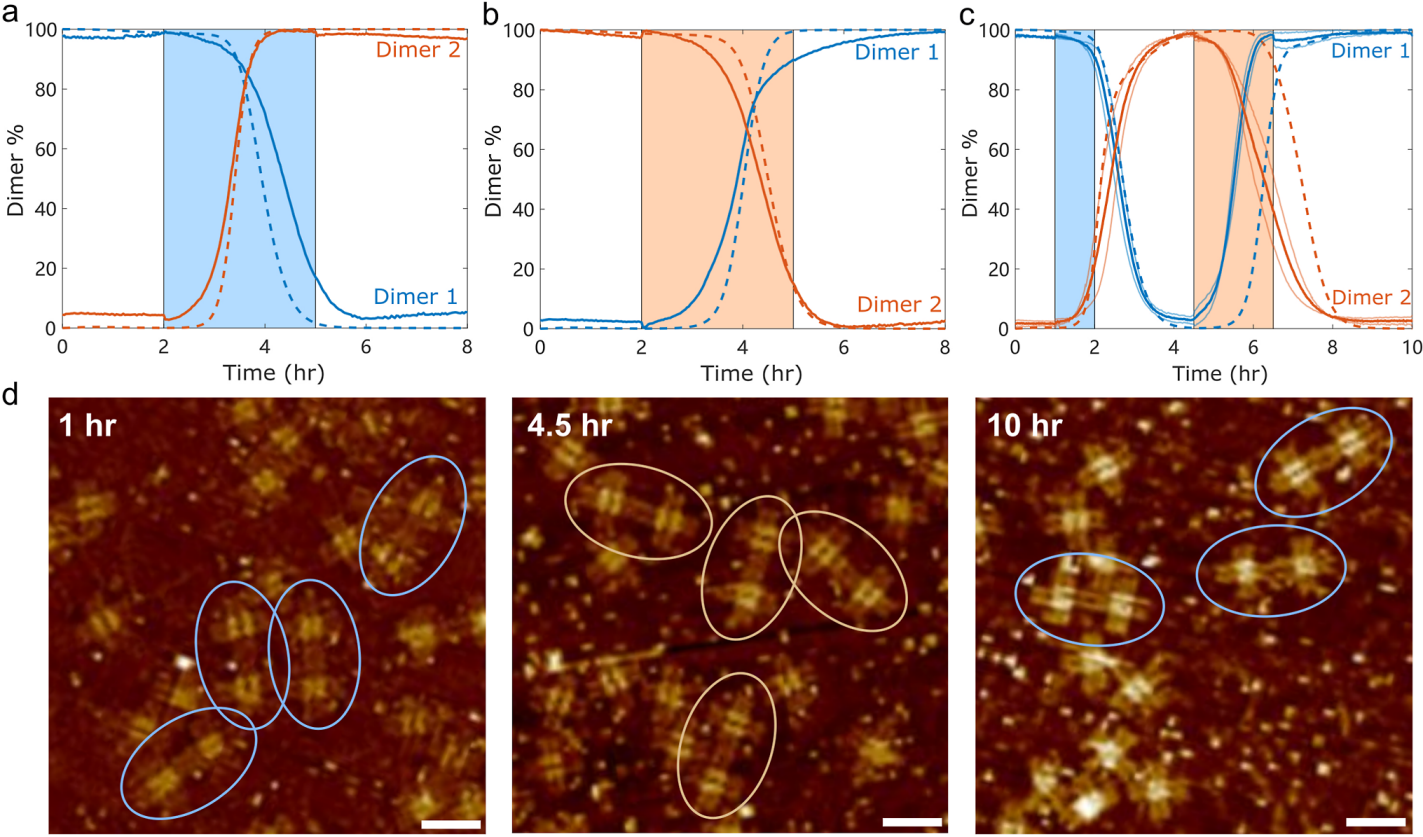
State switching by upstream genelets. (a,b) Dimer % of each tile pair during state switches from S1 to S2 via activation of Inducer Genelet G1 (a), from S2 to S1 via activation of Inducer Genelet G2 (b). The blue and orange domains indicate the time periods during which Inducer Genelet G1 or G2 is active, respectively. (c) The state could be switched multiple times (S1 to S2 to S1) by sequentially activating and deactivating the relevant Inducer Genelet. Solid lines represent the average of two measurements; thin lines represent two individual replicates; dashed lines represent simulations. (d) AFM images acquired at 1 h, 4.5 h, and 10 h verified a state switching from the initial state S1 (Dimer 1 with “ = = ” motif takes dominance) to S2 (Dimer 2 with “|| ||” motif takes dominance) and finally back to S1. Blue ovals indicate Dimer 1 and orange ovals indicate Dimer 2. Scale bar, 100 nm.

### Simulations of Other Dynamic Behaviors

These cross-tile pairs have the potential to be combined to produce a variety of dynamic behaviors. Inspired by the well-characterized feed-forward loop motif in both biological^48^ and synthetic gene regulatory networks,^35^ we simulated two network configurations using tile pairs that can either sustain oscillations^37,49^ or generate transient pulses.^50,51^ By selecting appropriate parameters, simulations predict that networks consisting of three or five tile pairs can exhibit persistent oscillations (Figure S24a,b). Furthermore, by changing the interactions between them, the same set of five tile pairs can show tunable temporal pulses in dimerization (Figure S24c).

## Conclusions

In this study, we demonstrate that the dynamic self-assembly of DNA origami tiles can be regulated by transcriptional modules whose activity is governed by spatial organization. This design establishes an autonomous feedback loop in which the nanostructure’s assembly state directly regulates the transcriptional reaction rate. Using programmable DNA and RNA strand displacement, we could control tile dimerization and implement feedback loops to create self-regulating systems. Combining two mutually inhibitory tile pairs, we constructed a dynamic bistable network that could be switched by either exogenous RNA inducers or upstream transcriptional circuits. Simulations further revealed that extended networks of cross-tile pairs can generate complex nonequilibrium temporal behaviors such as oscillations and pulses, highlighting the potential for constructing programmable, adaptive self-assembly systems.

Beyond DNA origami, this mechanism is conceptually applicable to other self-assembly platforms, presenting potential for future work in systems including DNA nanotubes,^6,7,38^ droplets,^52,53^ and colloids,^54,55^ through the core design principle that changes in local concentration upon assembly can modulate genelets activity. To ensure adaptability and precise regulation across these diverse environments, the kinetics can be tuned by regulating both the RNA production rate (by template design, such as changing the length of the activator strand) and the RNA degradation rate (by adjusting the RNase H concentrations), allowing the system to be precisely tailored for different applications. Moreover, such systems can be coupled with other dissipative processes, such as self-replication.^56,57^ For instance, RNA transcripts can be designed to influence subsequent generations, enabling the evolution of nanostructures toward controlled and programmable trajectories.

In addition to dynamically controlling self-assembly, the transcriptional modules developed in this work offer powerful tools for sensing, amplifying, and relaying molecular signals to DNA-based nanoscale materials and devices.^58,59^ These modules can be integrated into dynamic DNA nanodevices that reconfigure or move in response to RNA signals, translating biochemical inputs into mechanical outputs. In turn, the resulting structural changes can reorganize transcriptional components—such as repositioning genelets and activators—thereby generating new signals that drive subsequent motions. This feedback loop enables the design of autonomous, adaptive nanomachines with broad potential in biosensing,^60^ smart therapeutics,^61^ molecular robotics,^62^ and the development of complex artificial life systems.^63^

## Supplementary Material



## Notes

Experimental methods, DNA origami tiles design and sequences, DNA origami structural stability, transcription kinetic measurements, dimer formation and dissociation quantification, inducer strands design and sequences, genelets design and sequences, kinetic model, and simulation of other dynamic systems (PDF)
