# Supplementary material for: Dynamic Control of DNA Origami Self-Assembly by Transcriptional Modules
Source: J Am Chem Soc. 2026 Jan 7;148(2):2673–82. doi: 10.1021/jacs.5c18964 (PMC12833798; doi:10.1021/jacs.5c18964)
Supplement: Supplementary file 1 [file ja5c18964_si_001.pdf]

Supplementary Information

Dynamic Control of DNA Origami Self-assembly by Transcriptional Modules

Lei Zhang[1,], Ruojie Sha[2], Lev Bershadsky[1], Paul M Chaikin[1,*]*

[1]Department of Physics, New York University, New York, NY 100038, United States

[2]Department of Chemistry, New York University, New York, NY 100038, United States

*Lei Zhang: lz3202@nyu.edu

*Paul M Chaikin: chaikin@nyu.edu

1. Experimental Methods

1.1 Materials and Reagents

T7 RNA Polymerase (M0251S), RNAPol Reaction Buffer (B9012S), RNase H (M0297S) and Pyrophosphatase, Inorganic (yeast) (M2403S) were purchased from New England Biolabs. Ribonucleotide triphosphates were purchased from ThermoFisher Scientific. Agarose (A9539) was purchased from Sigma-Aldrich.

The single-stranded M13mp18 DNA genome was purchased from Bayou Biolabs. The remaining DNA strands and synthetic RNA blocker and inducer strands were purchased from Integrated DNA Technologies, Inc. The sticky-end staple strands were purified using denaturing PAGE. DNA strands with fluorophore or quencher were purified by HPLC. Other DNA strands underwent standard desalting but no purification. See Table S1, S2, S3, S4, S6, and S8 for sequences of staples on edge positions, transcriptional templates, blocker and inducer strands, reporter duplex strands, transcriptional template, and Inducer Genelet, respectively.

1.2 Formation of Individual DNA Origami Tiles and Dimers

Staple strands and M13mp18 DNA genome were mixed together in 1X TAE/Mg^{2+} buffer (40 mM Tris·HCl, 20 nM acetic acid, 2.5 mM EDTA, and 12.5 mM magnesium acetate, pH 8.0). The molar ratio M13mp18 DNA genome: staple strand was 1:8, and the final concentration of the mixture was 10 nM. The mixture was heated to 70 °C for 30 min and cooled to 20 °C at a rate of −7 °C/h in a thermocycling incubator. The resulting origami tiles were purified in 100 K Millipore Amicon Ultra 0.5-mL centrifugal filters using 1× TAE/Mg^{2+} for 3 times and then using 1x RNAPol Reaction Buffer twice. The stock origami tiles were stored under 4 °C.

When anneal DNA origami dimers, the individual left and right tiles were mixed, heated to 50°C for 30 min, and cooled to 20°C at a rate of −0.7°C/h.

1.3 Reporter and Genelets annealing and preparation

DNA duplex reporter was prepared by diluting the fluorophore-modified DNA strand of 5 μM per strand with its partially complementary quencher-modified DNA strand of 5.5 μM per strand (10% excessive) at a concentration in 1× NEB RNAPol reaction buffer. The genelets were prepared by template of 1 μM per strand with non-template strands of 1.1 μM per strand (10% excessive) at a concentration in 1× NEB RNAPol reaction buffer. The mixtures were heated to 70 °C, incubated for 30 min, then cooled down to 20 °C at a rate of -20 °C/hr.

1.4 Gel Electrophoresis

A 0.8% agarose gel was prepared and immersed in the 1× TAE/Mg^{2+} buffer inside the gel electrophoresis box. 9 μL samples were mixed with 1 μL nondenaturing tracking dye (1× TAE/Mg^{2+} buffer, 50% glycerol, and trace amount of bromophenol Blue and Xylene Cyanol FF). Gel Electrophoresis was run at 5 V/cm at 37 °C for 3 h. Then, the gel was immersed in the ethidium bromide solution 20 min for staining and rinsed with water to remove excess stain before imaging by Bio-Rad ChemiDoc.

1.5 AFM imaging

AFM imaging was performed in peakForce mode in air. 2 to 5 μL of sample was deposited on a clean mica surface (Ted Pella, Inc.) for 1 min. When characterizing the tiles self-assembly state, the sample was diluted 4- to 5-fold to a final concentration of approximately 5–6 nM before imaging. The mica was then washed with 50 to 70 μL of double-distilled H_2O three times and subsequently dried using compressed air. PeakForce in air mode was performed on the Nansocope V Multimode 8 scanning probe microscope (PeakForce QNM Software, ScanAsyst-HR accessory). Silicon nitride tips (ScanAsyst-Air; Bruker Nano, Inc.) were used for the Nanoscope V Multimode 8 SPM.

1.6 Fluorescence data acquisition

Unless otherwise stated, transcriptional rate measurements and dynamic assembly experiemnts were conducted at 37 °C in 1× NEB RNAP reaction buffer supplemented with $MgCl_2$ (at a final concentration of 30 mM Mg^{2+}); ribonucleotide triphosphates (ATP, UTP, CTP, GTP, at a final concentration of 3.75 mM each). Fluorescence readings were then taken by Biorad CFX96 Real Time PCR system.

2. Design of DNA origami tiles

The design and sequences of the cross-shaped origami tile staple strands is the same as the 'A origami tile' from Wenyan Liu et al.'s work[1] except those at edge positions. There are 6 DNA staple strands at edge positions on each edge. Three edges are poly-T strands and one binding edge with 4 sticky ends and 2 transcriptional modules. Design of tiles in this work was summarized in Figure S1. Sequences of those DNA staple strands at edge positions are summarized in Table S1 and Table S2.

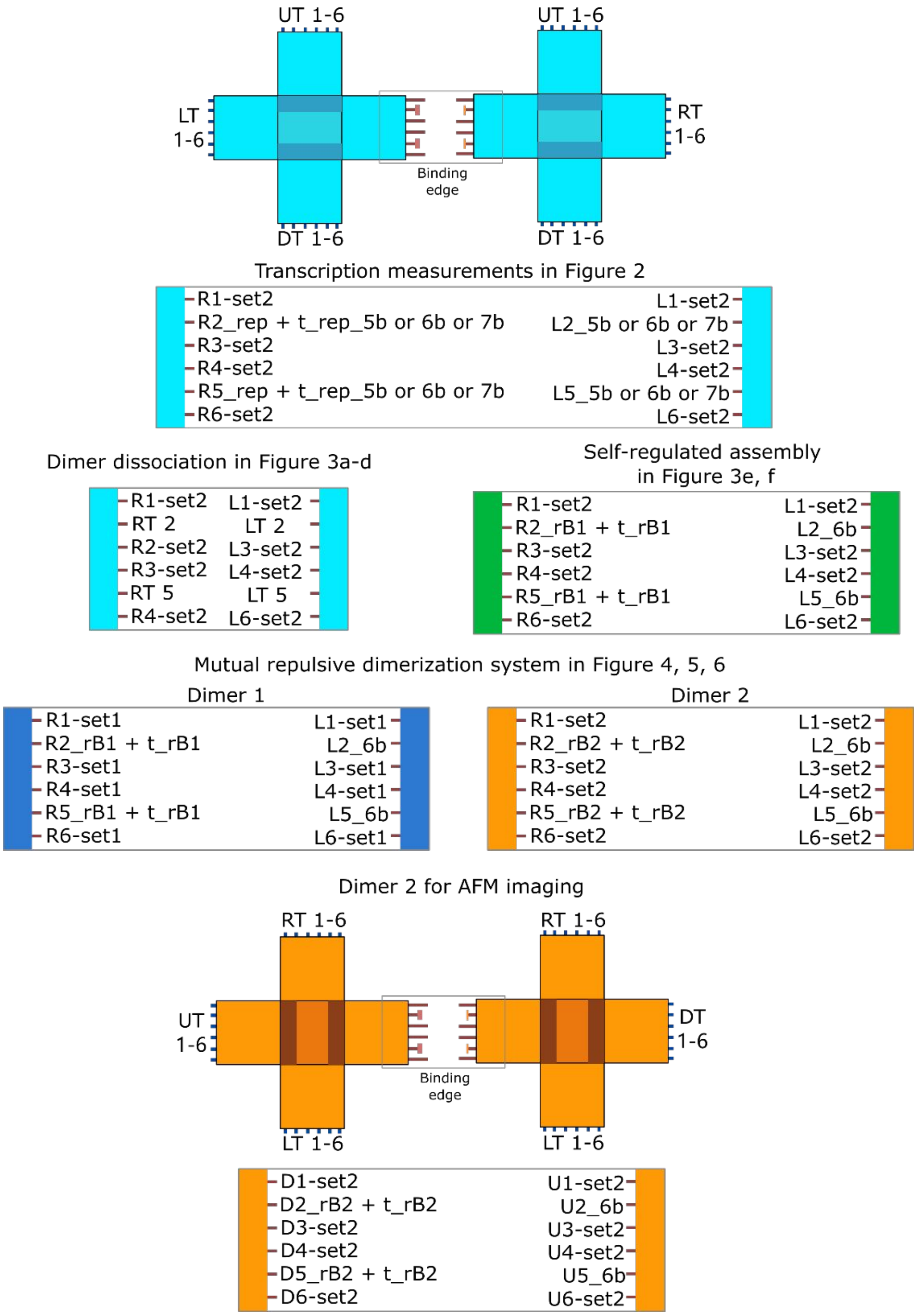

Figure S1. Desigen of tiles in each experiment. Three edges are poly-T strands. The binding edge is zoomed in to show strands details.

Table S1. Sequences of staple strands at tile edge positions. Sticky ends are colored orange. Toeholds are colored blue.

	Sticky ends
R1-set1	TAATTTGTGCCAGTGCGAGACA/iCy5/TGTTAAATAAGAATAAAGTGT GATAAATAAGGC
R3-set1	TAATTTGTGCCAGTGCGACCCTTAAATAAAGAAATTGCGTTAGCACG TAAAACAG
R4-set1	TAATTTGTGCCAGTGCGAGAGTTTATTCCTGATTATCAGAGCGGAAT TATCATCA
R6-set1	TAATTTGTGCCAGTGCGAACCTTACATTGGCAGATTCACCTGAAATG GATTATTT
L1-set1	/5IAbRQ/TGTCTCGCACTTTCCTGAACAAGAAAAATCAACAATAGAT AAG
L3-set1	AGGGTCGCACTTAATAATAAGAGCAAGAGAATTGAGTTAAGCCC
L4-set1	ACTCTCGCACTTGTTTGAGGGGACGACGAACCGTGCATCTGCCA
L6-set1	AGGTTCGCACTTAGCTGATTGCCCTTCACAGTGAGACGGGCAAC
R1-set2	CAGTTCACGTTTGACCACGACA/iCy3/TGTTAAATAAGAATAAAGTGT GATAAATAAGGC
R3-set2	CAGTTCACGTTTGACCACTGGCTAAATAAAGAAATTGCGTTAGCACG TAAAACAG
R4-set2	CAGTTCACGTTTGACCACCCCATTATTCCTGATTATCAGAGCGGAAT TATCATCA
R6-set2	CAGTTCACGTTTGACCACAGCCTACATTGGCAGATTCACCTGAAATG GATTATTT
L1-set2	/5BHQ_2/TGTCGTGGTCATTCCTGAACAAGAAAAATCAACAATAGA TAAG
L3-set2	GCCAGTGGTCATAATAATAAGAGCAAGAGAATTGAGTTAAGCCC
L4-set2	TGGGGTGGTCATGTTTGAGGGGACGACGAACCGTGCATCTGCCA
L6-set2	GGCTGTGGTCATAGCTGATTGCCCTTCACAGTGAGACGGGCAAC
D1-set1	TAATTTGTGCCAGTGCGAGACACGTTAATATTTTGTTAATATTTAAAT TGTAAA
D3-set1	TAATTTGTGCCAGTGCGACCCTATTAGATACATTTCGCTAGATTTAGT TTGACC

D4-set1	TAATTTGTGCCAGTGCGAGAGTATCAAAAAGATTAAGAAAGCAAAGCGGATTGC
D6-set1	TAATTTGTGCCAGTGCGAACCTGGATATTCATTACCCAATCTTCGACAAGAACC
U1-set1	TGTCTCGCACTAATAAGTTTATTTTGTCGCAAAGACACCACGG
U3-set1	AGGGTCGCACTAATTTACCGTTCCAGTGAAAGCGCAGTCTCTG
U4-set1	ACTCTCGCACTGGTTTAGTACCGCCACATCACCGTACTCAGGA
U6-set1	AGGTTCGCACTGAGGACTAAAGACTTTCGGCTACAGAGGCTTT
Poly T	
RT1	GTTAAATAAGAATAAAGTGTGATAAATAAGGCTTTTT
RT2	AAATCGTCGCTATTAAATAACCTTGCTTCTGTTTTTT
RT3	AAATAAAGAAATTGCGTTAGCACGTAAAACAGTTTTT
RT4	TATTCCTGATTATCAGAGCGGAATTATCATCATTTTT
RT5	TGCTGAACCTCAAATAATCTAAAGCATCACCTTTTTT
RT6	ACATTGGCAGATTCACCTGAAATGGATTATTTTTTTT
LT1	TCCTGAACAAGAAAAAATCAACAATAGATAAGTTTTT
LT2	TTGCACCCAGCTACAAAAGATTAGTTGCTATTTTTTT
LT3	AATAATAAGAGCAAGAGAATTGAGTTAAGCCCTTTTT
LT4	GTTTGAGGGGACGACGAACCGTGCATCTGCCATTTTT
LT5	CCCGGGTACCGAGGTCTCGACTCTAGAGGATCTTTTT
LT6	AGCTGATTGCCCTTCACAGTGAGACGGGCAACTTTTT
UT1	AATAAGTTTATTTTGTCGCAAAGACACCACGGTTTTT
UT2	TGTAGCGCGTTTTCATGCCTTTAGCGTCAGACTTTTT
UT3	AATTTACCGTTCCAGTGAAAGCGCAGTCTCTGTTTTT
UT4	GGTTTAGTACCGCCACATCACCGTACTCAGGATTTTT
UT5	ACTAAAGGAATTGCGAAGAATAGAAAGGAACATTTTT
UT6	GAGGACTAAAGACTTTCGGCTACAGAGGCTTTTTTTT
DT1	CGTTAATATTTTGTTAATATTTAAATTGTAAATTTTT
DT2	TGAGTAATGTGTAGGTTTTTAAATGCAATGCCTTTTT

DT3	ATTAGATACATTTCGCTAGATTTAGTTTGACCTTTTT
DT4	ATCAAAAAGATTAAGAAAGCAAAGCGGATTGCTTTTT
DT5	ATAACGCCAAAAGGAACAACTAATGCAGATACTTTTT
DT6	GGATATTCATTACCCAATCTTCGACAAGAACCTTTTT

Table S2. Sequences of transcriptional modules.

Transcription measurements	
R2_rep	AAATCGTCGCTATTAAATAACCTTGCTTCTGTTTTTTTTTAATAC GACTCACTATAGGGAGACAGATTAACCAGACAGTGAC
R5_rep	TGCTGAACCTCAAATAATCTAAAGCATCACCTTTTTTTTTAATAC GACTCACTATAGGGAGACAGATTAACCAGACAGTGAC
L2_5b	TATTATTTTTTTTGCACCCAGCTACAAAAGATTAGTTGCTATT
L2_6b	GTATTATTTTTTTTGCACCCAGCTACAAAAGATTAGTTGCTATT
L2_7b	CGTATTATTTTTTTTGCACCCAGCTACAAAAGATTAGTTGCTAT T
L5_5b	TATTATTTTTTTCCCGGGTACCGAGGTCTCGACTCTAGAGGATC
L5_6b	GTATTATTTTTTTCCCGGGTACCGAGGTCTCGACTCTAGAGGATC
L5_7b	CGTATTATTTTTTTCCCGGGTACCGAGGTCTCGACTCTAGAGGAT C
t_rep_5b	GTCACTGTCTGGTTAATCTGTCTCCCTATAGTGAGTCG
t_rep_6b	GTCACTGTCTGGTTAATCTGTCTCCCTATAGTGAGTC
t_rep_7b	GTCACTGTCTGGTTAATCTGTCTCCCTATAGTGAGT
Mutual repulsive dimerization system	
R2_rB2	AAATCGTCGCTATTAAATAACCTTGCTTCTGTTTTTTTTTAATAC GACTCACTATAGGGAGATCGCACTGGCACAAATTACTATG
R5_rB2	TGCTGAACCTCAAATAATCTAAAGCATCACCTTTTTTTTTAATAC GACTCACTATAGGGAGATCGCACTGGCACAAATTACTATG
t_rB2	CATAGTAATTTGTGCCAGTGCGATCTCCCTATAGTGAGTC
R2_rB1	AAATCGTCGCTATTAAATAACCTTGCTTCTGTTTTTTTTTAATAC GACTCACTATAGGGAGAGTGGTCAAACGTGAACTGAGTCG
R5_rB1	TGCTGAACCTCAAATAATCTAAAGCATCACCTTTTTTTTTAATAC GACTCACTATAGGGAGAGTGGTCAAACGTGAACTGAGTCG

t_rB1	CGACTCAGTTCACGTTTGACCACTCTCCCTATAGTGAGTC
U2_6b	GTATTATTTTTTTTGTAGCGCGTTTTCATGCCTTTAGCGTCAGAC
U5_6b	GTATTATTTTTTTACTAAAGGAATTGCGAAGAATAGAAAGGAAC A
D2_rB2	TGAGTAATGTGTAGGTTTTTAAATGCAATGCCTTTTTTTTAATAC GACTCACTATAGGGAGATCGCACTGGCACAAATTACTATG
D5_rB2	ATAACGCCAAAAGGAACAACTAATGCAGATACTTTTTTTTAATA CGACTCACTATAGGGAGATCGCACTGGCACAAATTACTATG

Table S3. Sequences of blocker and inducer strands.

dB1_no t	TCGCACTGGCACAAATTA
dB1_5t	CTATGTCGCACTGGCACAAATTA
dB1_3t	TCGCACTGGCACAAATTA CTATG
dB2	GTGGTCAAACGTGAACTGAGTCG
dI1_5t	TAATTTGTGCCAGTGCGACATAG
dI1_3t	CATAGTAATTTGTGCCAGTGCGA
rB1	UCGCACUGGCACAAAUUACUAUG
rI1	CAUAGUAAUUUGUGCCAGUGCGA
rB2	GUGGUCAAACGUGAACUGAGUCG
rI2	CGACUCAGUUCACGUUUGACCAC

3. Check DNA origami structures during transcription

T7 RNA polymerase has been reported to nonspecifically bind and transcribe a variety of DNA sequences[2], which could cause undesired deformation of disassembly of DNA nanostructures[3, 4]. Thus, the first step is to check if DNA tiles can survive and keep function with T7 RNAP. We examine the DNA origami structure by running agarose gels. When mixture of DNA origami dimer and monomers were incubated with T7 RNAP, non-specific transcription can cause clustering of DNA origami tiles (Lane 3 in Figure S2a). With RNase H that can degrade unwanted RNA, the bands were restored (Lane 4 and 5 in Figure S2a). In Lane 4, RNase H is added together with T7 RNAP, i.e., transcription and RNA degradation happen together. In Lane 5, RNase H is added just before running the gel. This means that even unwanted clusters of DNA origami can form during transcription, the overall structure of DNA origami can keep good state. Incubation with RNase H for short time (~15 min) is enough to degrade the 'linker RNA' from promoter-independent transcription and recover the DNA origami tiles.

We also took AFM images of the DNA origami tiles and found that they could keep in good shape during incubation for more than 20 hr with T7 RNAP and RNase H (Figure S2b). Particles in the background are enzymes including T7 RNAP and RNase H since AFM image of transcriptional solution even without DNA origami also showed these particles (Figure S2c). We also check the structures of dimers. 5 nM each monomers were mixed and incubated with T7 RNAP and RNase H for 20 hr. AFM images were taken The tile dimer can also survive after incubation with T7 RNAP and RNase H (Figure S2d), indicating that the sticky ends in this work can keep hybridization during transcription.

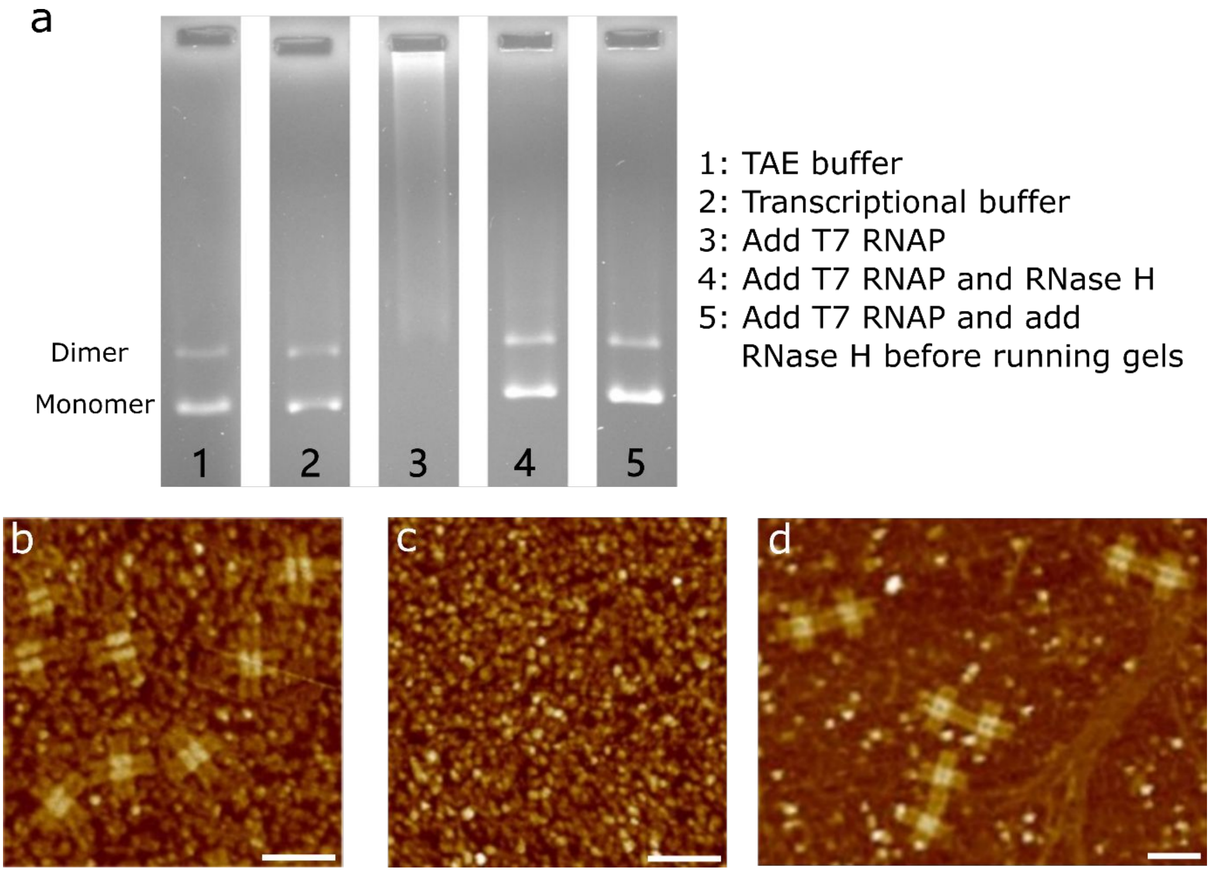

Figure S2. DNA origami in this work can keep good shape with T7 RNAP. (a) Non-denaturing agarose gels of mixtures of monomers and dimers during different conditions. (b) AFM image of tiles after incubation with T7 RNAP and RNase H for 20 hr. (c) AFM image of transcriptional solution with T7 RNAP and RNase H (no DNA origami). (d) AFM images of tile dimers after incubation with T7 RNAP and RNase H for 20 hr. Scale bars, 100 nm.

4. **Characterization of transcription rates of transcriptional modules.**

We measured transcription rates of transcriptional modules with 5, 6 and 7-base activator strands in monomer and dimer state. M1 indicates tile with genelets and M2 indicates tile with activator strands. If tiles have 4 pairs of sticky ends, they can bind to Dimer state; otherwise, the binding with genelets and activator strands are too weak, so they will stay Monomer state (Figure S3a). We used a DNA duplex reporter to quantify the amount of RNA produced by tiles in both Dimer and Monomer state (Figure S3b). Sequences of this DNA reporter duplex were summarized in Table S4. The components in these experiments were summarized in Table S5.

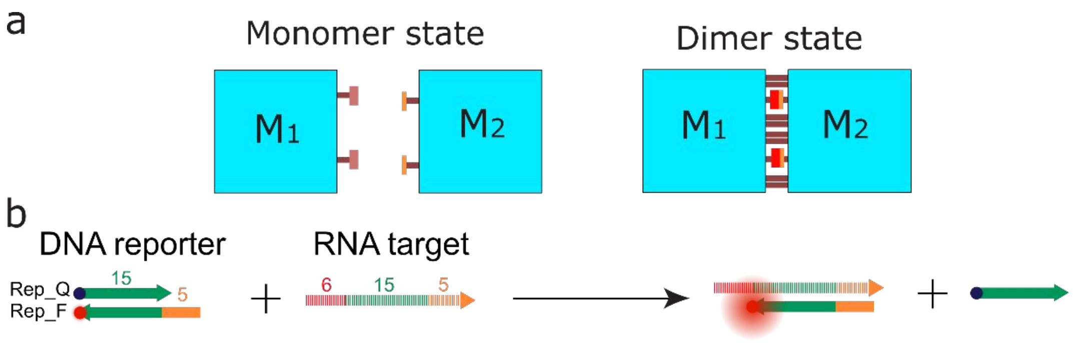

Figure S3. Transcription rate measurement. (a) M1 and M2 will bind with each other to form a dimer if they have 4 pairs of sticky ends; otherwise, they will stay in monomer state. (b) A DNA duplex reporter is used to quantify the transcription rates. The numbers next to a sequence domain specify the sequence domain's length in number of nucleotides.

Table S4. Sequences of DNA reporter duplex.

Rep_Q	/5IAbRQ/CAGATTAACCAGACA
Rep_F	GTCACTGTCTGGTTAATCTG/3TEX615/

Table S5. Components for transcriptional modules' activity characteristics.

**Component**	**Concentration**
M1	5 nM
M2	5 nM
Reporter	50 nM
T7 RNAP	2 U/uL
RNase H	0.005 U/uL
YIPP	0.002 U/uL

When measuring transcription rates by DNA reporter duplex, initial fluorescence values of were measured for around 10 min before T7 RNAP, RNase H and YIPP were added to initiate the reactions. At the end of each measurement, excessive amount of DNA strand fully complementary to strand Rep_F was mixed at a final concentration of 1 µM to a maximum

fluorescence value. The concentration of unquenched reporter was then obtained from measured fluorescence value (F) using Eq. 1. F_{min} and F_{max} indicate the minimum and maximum fluorescence value over the whole measuring process. $[Reporter]_0$ is the original reporter duplex concentration that is 50 nM unless otherwise stated.

$$[Unquenched\ reporter] = [Reporter]_0 \times \frac{F - F_{min}}{F_{max} - F_{min}} \qquad (Eq.1)$$

The transcription rate is expected to proportional with the dimer fraction, as only dimers provide active genelets for RNA production. To test this, we mixed 5 nM tiles containing genelets with 0–5 nM tiles carrying the activator strand, thereby generating samples with increasing dimer percentages. Upon addition of T7 RNAP, we measured RNA production rates (Figure S4) and found that they increased proportionally with the dimer fraction, i.e., with the concentration of active genelets. Transcription rates in Figure 2e were calculated using the [Unquenched reporter] at 30 min by $\frac{[\text{Unquenched reporter}]_{30\,min}}{30\,min}$.

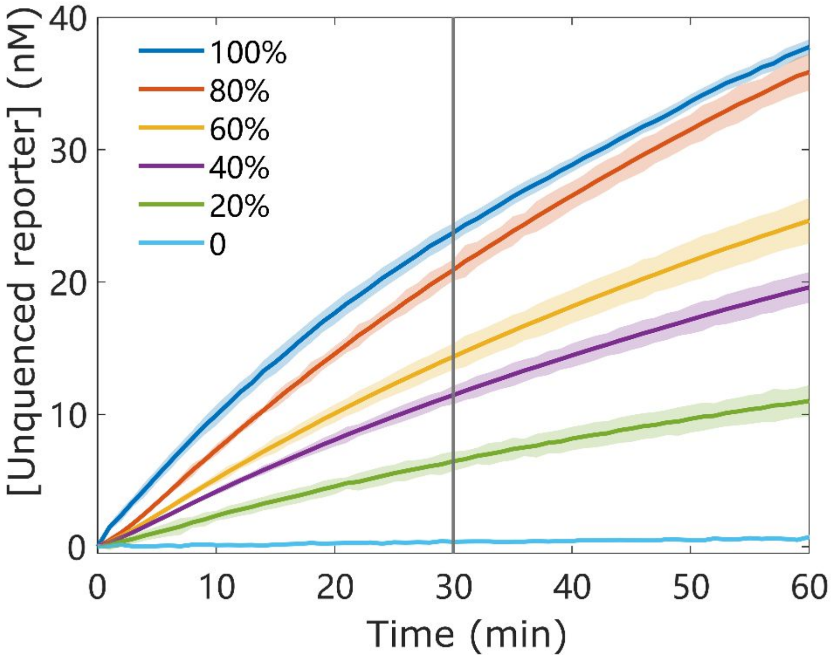

Figure S4. RNA transcription rate with different dimer percentages. Averages transcription rates were calculated by [Unquenched reporter] at 30 min. Shaded regions represent standard variations of 3 measurements.

5. Quantify dimer percentages from fluorescence quenching data and running agarose gel

We compared the results of dimer formation from fluorescence measurements with those from running gel to check if fluorescence can also give reliable results. 5 nM of tile with fluorophore (M_1) was mixed with 0, 1, 2, 3, 4 and 5 nM of another tile with quencher (M_2). Each sample was divided into two samples for agarose gel analysis and fluorescence measurements, respectively. Here, $Dimer\%$ is defined as the percentage of in M_1 dimer state.

When quantifying the dimer percentage by running gels, the intensity of upper gel bands (dimer) was compared to the total intensity of the bands (dimer and monomer) in the entire lane (Figure S5). The area of dimer band (S_D) and monomer band (S_M) can be quantified by Image J and Dimer% can be calculated by Eq. 2.

$$Dimer\% = \frac{S_D/_2}{S_D/_2 + S_M} \times 100\% \qquad (Eq.2)$$

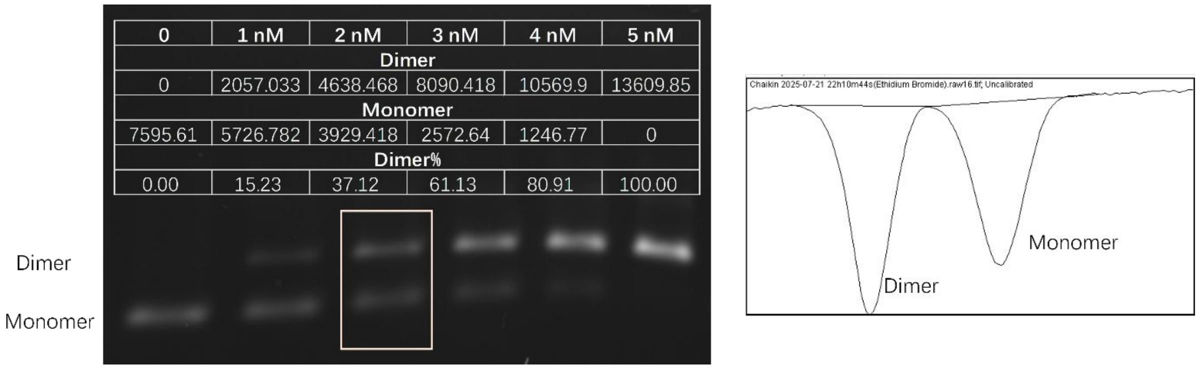

Figure S5. Quantify Dimer% by running non-denaturing agarose gel at 37℃. 5 nM M_1 was mixed with 0, 1, 2, 3, 4 and 5 nM M_2. The area of dimer band and monomer band using the ImageJ, "Plot lanes" function. Plot of the gel intensity over the width for '2 nM' sample was shown as demonstration. Dimer% can be calculated by Eq. 2.

Fluorescence measurements can also characterize Dimer%. Fluorophore is attached to sticky ends on M_1 and quencher is attached to sticky ends on M_2 (Figure S6a). Without M_2, M_1 itself gives the maximum fluorescence value ($I_{0\%}$), indicating Dimer% as 0%. With the addition of M_2, more fluorophore is quenching, leading to low fluorescence (Figure S6b). No apparent band for monomer was detected when 5 nM M_2 was added, indicating 100% yield. Thus, we can consider the Dimer% corresponding to the minimum fluorescence value ($I_{100\%}$) as 100%. Dimer% can be calculated as Eq. 3.

$$Dimer\% = \frac{I_{0\%} - I}{I_{0\%} - I_{100\%}} \times 100\% \qquad (Eq.3)$$

Dimer% results from running gels and measuring fluorescence both have linear relationship with M_2 concentrations (Figure S6c). So, we can track the kinetics of dimer formation and dissociation by measuring fluorescence.

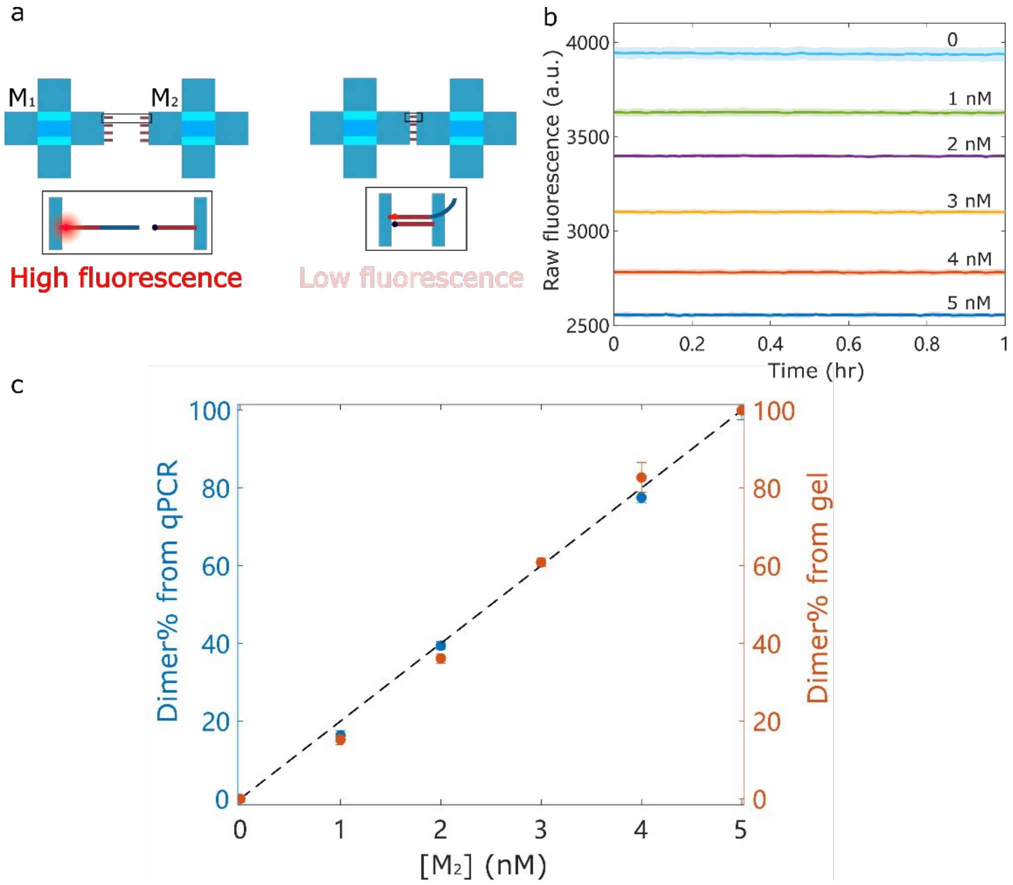

Figure S6. Quantify Dimer% by measuring fluorescence by qPCR. (a) A fluorophore and a quencher are incorporated into the complementary sticky ends of two tiles to monitor the dimerization by which fluorescence is quenched by closing quencher. (b) Raw fluorescence values of mixtures of 5 nM M_1 with different concentrations of M_2. Shaded regions represent maximum and minimum values. (c) Both running gels and measuring fluorescence give consistent measurements of dimer percentages. Error bars represent maximum and minimum values (Dimer% from qPCR) and standard deviations of 3 measurements (Dimer% from gel).

6. Transcription-induced dissociation

We investigated whether blocker strands could be RNA transcribed in situ. We designed a transcriptional template with promoter (pink domain) and downstream sequence (brown and blue domains) to produce the target RNA strands as blocker (rB) to trigger dimer dissociation (Figure S7).

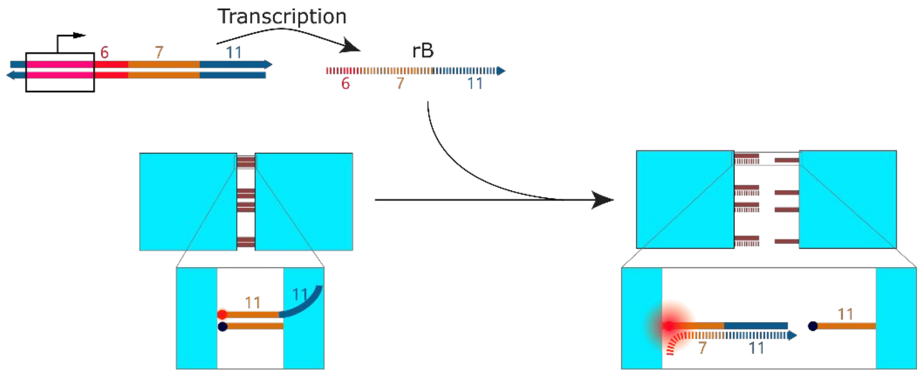

Figure S7. *In vitro* transcription directed dimer dissociation. The numbers next to a sequence domain specify the sequence domain's length in number of nucleotides.

Table S6. Sequences of transcriptional template for transcription-induced dissociation.

nt_rB1	TAATACGACTCACTATAGGGAGACAGTTCACGTTTGACCAC
t_rB1_full	GTGGTCAAACGTGAACTGTCTCCCTATAGTGAGTCGTATTA

Table S7. Components for transcription-induced dimer dissociation measurements.

**Reagent**	**Concentration**
M_1	5 nM
M_2	7 nM
G_rB1	0.5 nM
T7 RNAP	2 U/uL
RNase H	0.005 U/uL
YIPP	0.002 U/uL

7. Design of Inducer strands

We sought to develop an approach to switch the state of the mutually repulsive self-assembly system (Figure 5) by RNA inducers (rI) that prevent RNA blockers (rB) from acting downstream. For example, when the current system is in State 1 (Dimer 1 takes dominance and Dimer 2 is dissociated), inducer rI1 can inhibit blocker rB^1, thereby promoting Dimer 2 formation. The rB^2 transcribed by Dimer 2 subsequently dissociates Dimer 1 into monomers, switching the system into State 2.

Besides inhibiting free RNA blockers in solution through nucleic acid hybridization, the inducer strands can also remove blocker strands from sticky ends by toehold-mediated strand displacement reaction. Otherwise, those blocker strands will be removed only by degradation by RNase H (Figure S8a). The rate can be modeled as

$$r = -\,k_d[S{:}rB] \qquad (Eq.4)$$

The relationship between yield and time can expressed as

$$t = \frac{\ln\left(\frac{1}{1-yield}\right)}{k_d} \qquad (Eq.5)$$

The value of k_d in this work is fitted as 1.1×10^{-4} s^{-1}. This means that the time to remove 90% of RNA blocker by RNase H is $\frac{\ln\left(\frac{1}{1-0.9}\right)}{1.1\times10^{-4}s^{-1}}$ = 2.1×10^{4} s ≈ 6 hr. This slow rate limits the construction of dynamic systems. Besides, increasing inducer strands concentration will not increase degradation significantly.

Thus, we include an overhang on the blocker strands as toehold that allows the inducer strands to remove the blocker strands from sticky ends via strand displacement reaction. The rate of strand displacement reaction can be modeled as

$$r = -\,k_{dis}[S{:}rB] \times [rI] \qquad (Eq.6)$$

Using the fitted value of k_{dis} in this work, we simulated the kinetics of removing blocker strands via strand displacement reaction (Figure S8b). It is much faster than degradation and its rate can be accelerated by increasing inducer strands concentration. Thus, we chose the design of including an overhang on the blocker strands as toehold.

This toehold can be added at the 5' or 3' end of the blocker strands, corresponding to 3' or 5' end of the inducer strands (Figure S8c). We added 50 nM blocker strand with 5' or 3' toehold to 5 nM dimer to approach monomer state. Then, 100 nM corresponding inducer was added to induce the monomers rebinding to dimers. We found that 3' end design enables dimer to recover at a higher rate (Figure S8d). Thus, we put toehold at 3' end of the blocker strands in the following experiments.

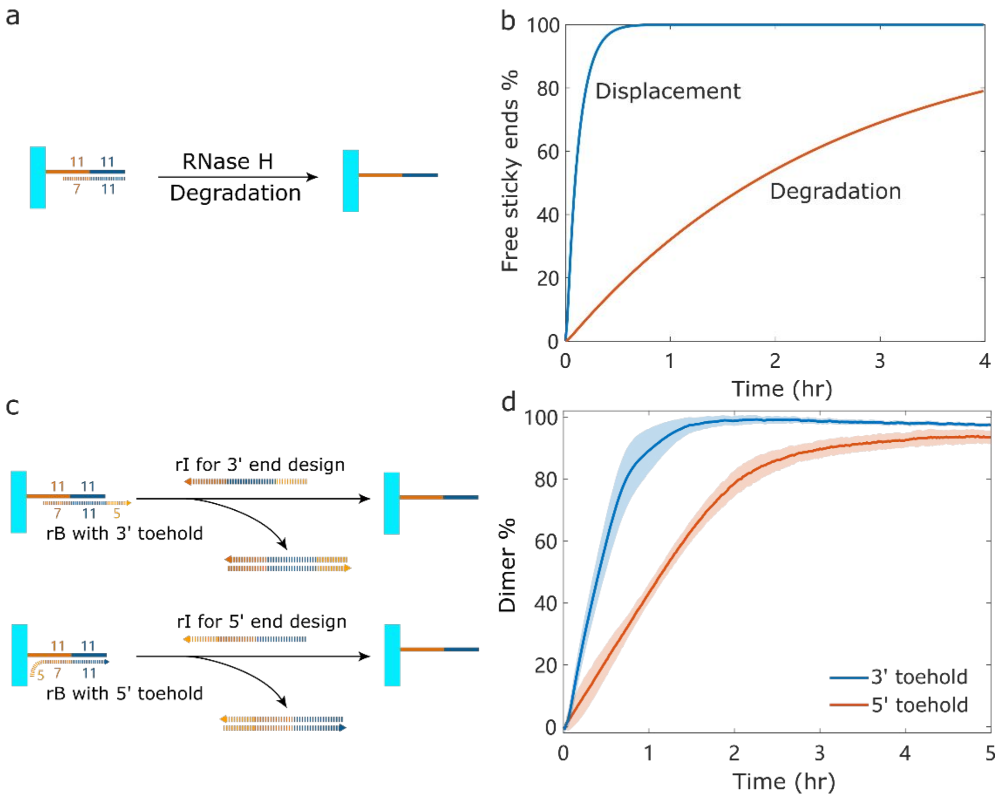

Figure S8. Design of inducer strands. (a) If the inducer strands cannot remove the blocker strands bound to sticky ends by toehold-mediated strand displacement reaction, those blocker strands will be removed only by degradation by RNase H. (b) Removing blocker strands via strand displacement reaction is much faster than degradation. (c) The toehold can be added at the 5' or 3' end of the blocker strands. (d) 3' end design can help the recovery of dimer more efficient. Shaded regions represent 3 measurements. The numbers next to a sequence domain specify the sequence domain's length in number of nucleotides.

8. Quantify kinetics of dimer formation and dissociation by fluorescence measurements

8.1 Default procedure

We measured fluorescence to quantify the kinetics of dimer formation and dissociation. The amount of Dimer is described by Dimer% (the percentage of tiles in the dimer state) that can be obtained from fluorescence data using Eq. 7.

$$Dimer\% = 100\% \times \frac{F_{0\%} - F}{F_{0\%} - F_{100\%}} \qquad (Eq.7)$$

$F_{100\%}$ represents the fluorescence value when all tiles are in the dimer state. In each assay, two monomer tiles were mixed and incubated for ~1 hour until dimerization was complete. In the absence of blocker strands, no visible monomer bands were observed in the gel images (Figure S5, 5nM), indicating that the dimer yield was effectively 100%. Therefore, $F_{100\%}$ was determined by measuring the fluorescence before adding RNA blockers. $F_{0\%}$ represents the fluorescence when all tiles are in the monomer state. This value was obtained by adding an excess of DNA blocker strands (1 μM) to each assay at the end of the experiment.

Here, we give an example (Figure 5b, 300 nM) of normalization of fluorescence data (Figure S9) in mutually repulsive self-assembly system. Fluorescence using corresponding filter was measured before adding 50 nM rB1 to set the initial conditions. After measurements, 1 nM dB1 and dB2 were added to acquire maximum fluorescence values. $F_{0\%}$ and $F_{100\%}$ were determined by the minimum and maximum fluorescence over an entire experiment.

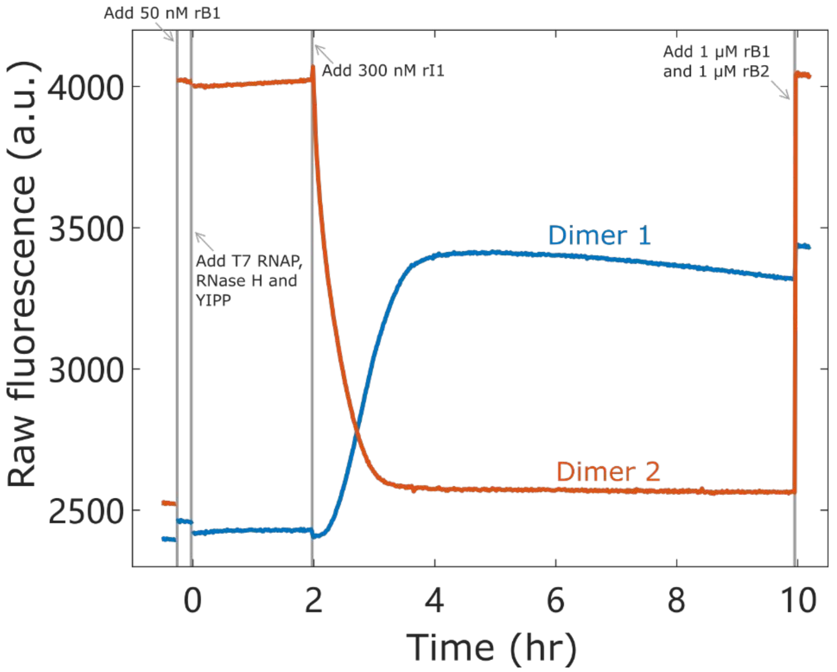

Figure S9. Example of quantifying kinetics of dimer formation and dissociation by fluorescence measurements.

8.2 Analyzing fluorescence during multiple times state switching

For the experiments involving twice state switch (Figure 6c), the fluorescence data was analyzed in two parts. This approach was to account for a significant decrease in the absolute maximum fluorescence intensity over the course of the experiment. Specifically, the maximum fluorescence value for Dimer 2 (Cy5-labeled) observed at the end of the experiment, after the addition of excessive dB, was approximately 5% lower than the initial maximum fluorescence value obtained after the system was initialized with rB1 (Figure S10a). This decline in maximum fluorescence intensity is likely due to dilution and DNA binding to the pipette tips when adding enzymes and strands over the course of the experiment.

This phenomenon is consistent with observations in previous genelet circuit studies[5, 6]. To address this shift in the absolute fluorescence range, we employed the same two-part normalization strategy utilized in previous work by Schaffter et al.[5]: the data corresponding to the *S1* to *S2* switch was normalized using the maximum fluorescence value ($F_{max,1}$) obtained before adding enzymes and the data corresponding to *S2* to *S1* switching was normalized using the maximum fluorescence value ($F_{max,2}$) obtained after adding dB1 (Figure S10b). The results from this two-part normalization method align well with the AFM images where no Dimer 2 was found (Figure S17, Table S11).

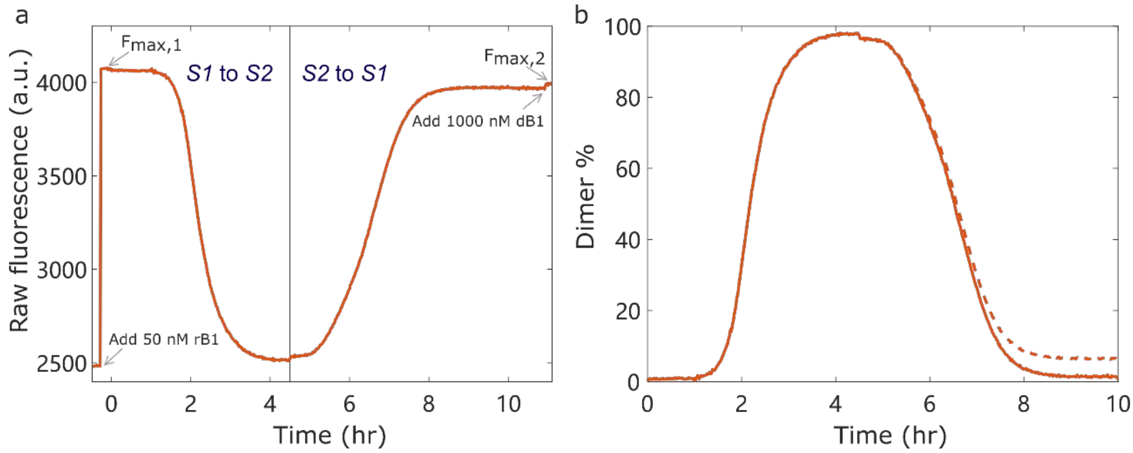

Figure S10. Example of quantifying kinetics of dimer formation and dissociation during multiple times state switching. (a) Raw fluorescence values for Dimer 2 in Figure 6c. The data corresponding to switching from *S1* to *S2* was normalized using the maximum fluorescence value obtained before adding enzymes ($F_{max,1}$) and the data corresponding to switching from *S2* to *S1* was normalized using the maximum fluorescence value obtained after adding dB1 ($F_{max,2}$). (b) Comparison between using one-part method (dashed line) and two-part method (solid line). This two-part method can address the shift in the absolute fluorescence range during experiments.

8.3 Effect of photobleaching

Photobleaching can cause loss in fluorescence intensities, which could impact the accurate quantification of Dimer%, particularly for tiles in monomer state (corresponding to high fluorescence intensity). To evaluate this effect, we measured the fluorescence intensity loss of dye Cy3 and Cy5 during fluorescence measurement. The fluorescence reading proceeded

every minute. After taking readings every minute for 15 hr (900 fluorescence readings), we observed a minimal loss of ~2% for Cy3 dye and ~3% for Cy5 dye (Figure S11).

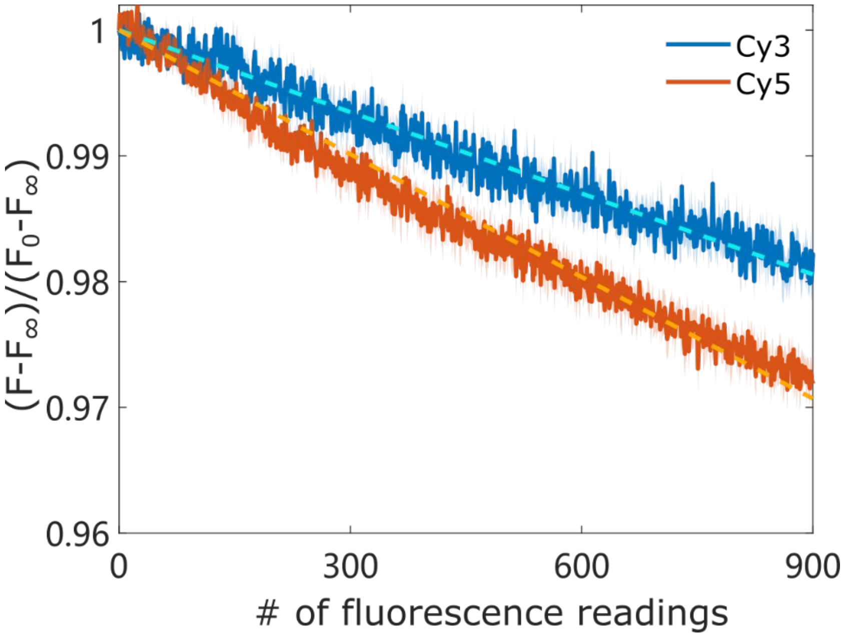

Figure S11. Fluorescence loss of dye Cy3 and Cy5 during fluorescence measurements. The fluorescence reading was taken every minute for 15 hr. Solid lines represent experimental results. Shaded regions represent standard variations of 3 measurements. Dashed lines represent simulations.

Under a constant light absorption, the fluorescence intensity will decrease over time following an exponential decay law: $F - F_\infty = (F_0 - F_\infty) \times e^{-K\times N}$, where F is the measured fluorescence intensity, F_∞ is final, background fluorescence that is set as the fluorescence of buffer, F_0 is the initial fluorescence intensity, K is the bleaching rate constant, and N is the number of fluorescence readings. The bleaching rate constant for Cy3 and Cy5 dye was fitted as 2.18×10^{-5} and 3.30×10^{-5}, respectively.

We next evaluated the effect of photobleaching on analysis of fluorescence measurements. The impact of photobleaching can be corrected by converting measured fluorescence into original fluorescence by $F_0 = F_\infty + (F - F_\infty)/e^{-K\times N}$. We compared the Dimer% results for the tests of maintaining bistable state (Figure 4b) using both the photobleaching-corrected and the raw measured fluorescence intensities. For the dominant dimers, there is no distinguishable difference. For the tile pairs mainly in monomer state, photobleaching caused a slight overestimation of the Dimer%: the difference after 12 hr measurement was ~1% for Dimer 1 (Cy3) and ~2% for Dimer 2 (Cy5) (Figure S12). Given this minimal impact, we did not apply the correction when interpreting the measured fluorescence intensities in the dynamic assembly experiments.

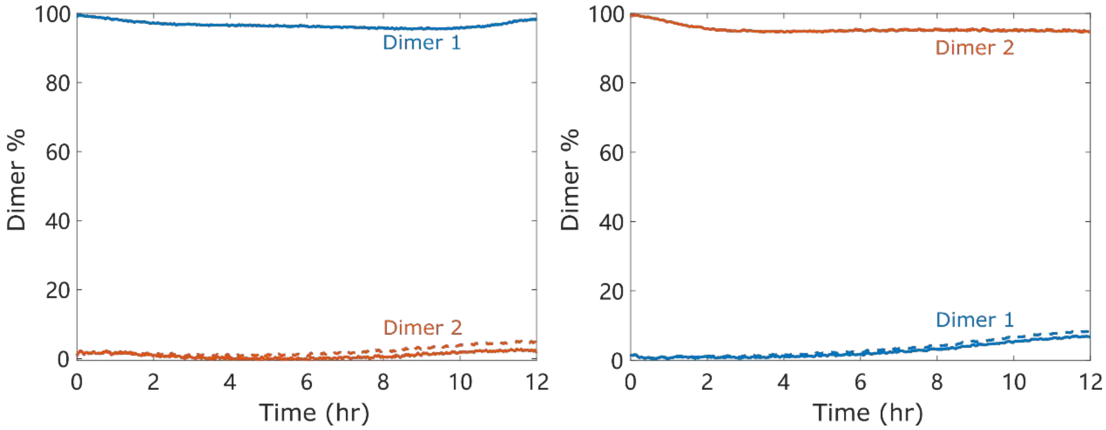

Figure S12. Dimer% results with photobleaching correction (solid lines) and using raw measured fluorescence intensities (dashed lines).

8.4 Discussion of slight increases in Dimer% of non-dominant tile pairs

In the analysis of the bistable self-assembly system, the tile pairs mainly in monomer states exhibited a slight, consistent fluorescence decrease over 12 hours, corresponding to a minor increase in the Dimer percentage over time (reaching 4%–7% in the dimer state at 12 hr; Figure 4b). This time-dependent fluorescence decrease is consistent with observations in other studies[5, 6] involving multi-state biological networks that utilize fluorescent dyes to monitor distinct states. While photobleaching contributes partly to fluorescence decrease (Figure S12), the observed time-dependent decrease is significantly greater than photobleaching alone. Here, we discussed another possible reason causing this phenomenon: this slight fluorescence decrease might be due to transient interactions between the dyes and accumulated RNA transcripts. Although these RNA transcripts would not have stable interactions with the blocked sticky ends with dyes, they might form transient stacking or very short, unstable hybrid (2-3 base pairs). This transient interaction can bring the dye into close proximity to the RNA's nucleobases, causing fluorescence decrease due to Photoinduced Electron Transfer (PET)[7].

We had a qualitative test of effect of PET on measured fluorescence intensities. 200 nM and 500 nM random RNA strand was added to 5 nM tiles with Cy3 label. There is a significant decrease in fluorescence intensities after adding the random RNA strand (Figure S13). It should be noted that the real case is much more complicated: there has been already a lot of DNA and free NTPs in the system, which means that PET has already affected the accuracy of fluorescence measurements; PET effect depends on the sequence and length of the RNA greatly. Thus, it is not easy to correct this effect. Luckily, this effect only has less than 5% shift in Dimer% in our bistable until 12 hr. Therefore, this phenomenon does not substantially compromise the fundamental interpretation of the kinetic and assembly results from the fluorescence measurements.

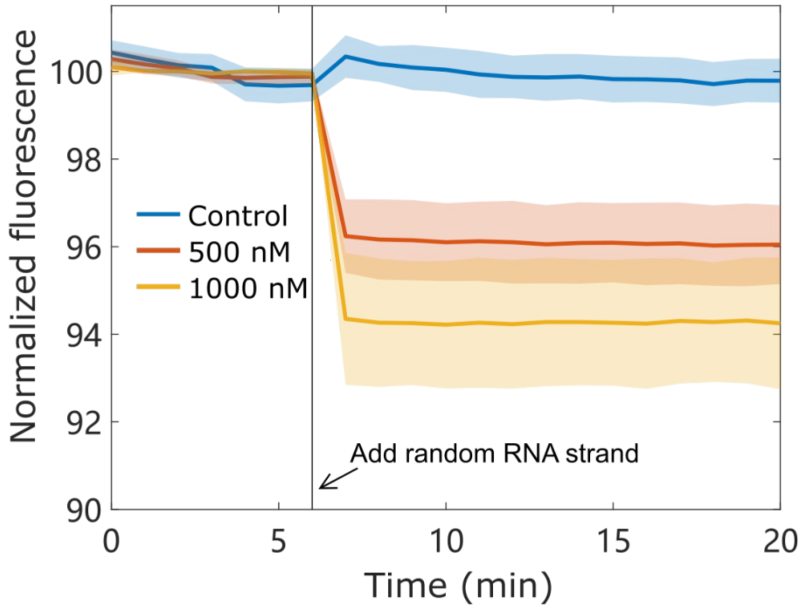

Figure S13. Fluorescence decreases due to Photoinduced Electron Transfer (PET). There is a significant decrease in fluorescence intensities after adding the random RNA strand. 'Control' test means adding same volume of buffer to isolate the fluorescence change from any potential effect caused by volume dilution. Solid lines represent average of 4 measurements. Shaded regions represent standard variations of 4 measurements.

9. Supplementary state switching results

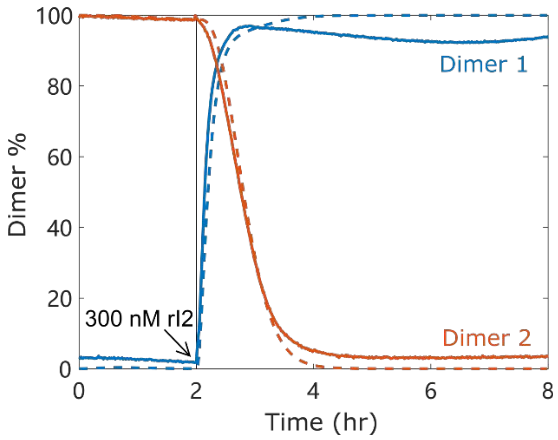

Figure S14. The state switch from *S2* to *S1* after incubation for 2 hr is also available by adding 300 nM rI2.

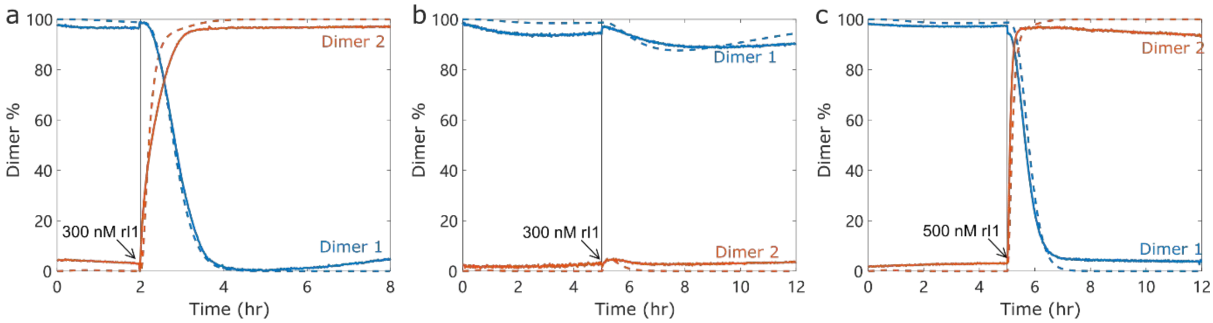

Figure S15. The required amount of RNA inducers depends on the incubation time following T7 RNAP addition. Although 300 nM rI1 can switch the state from *S1* to *S2* after 2 h of incubation (a), the same amount becomes insufficient after 5 h (b). In this case, 500 nM rI1 is required to achieve state switching (c).

10. Design of Inducer Genelets

We designed the inducer modules based on the HPC5 nodes in the work by Schaffter, S.[6]. Transcription can occur only when the activator is bound and completes the promoter domain. An RNA repressor sequesters the DNA activator to inhibit transcription (Figure S16). We utilized the sequences in G1 (still designated as G1' in this work) and G4 (designated as 'G2' in this work) and designed the template node that produces the inducer strands in our work. Sequences of Inducer Genelets were shown in Table S8.

Table S8. Sequences of Inducer Genelets

G1_nt	TCCTTCCATGCACGCCAAACCGTGGCGACGTAATACGACTCACTATAG GGAGATTCGTCTCCCCGACTCAGTTCACGTTTGACCAC
G1_t	GTGGTCAAACGTGAACTGAGTCGGGGAGACGAATCTCCCTATAGTGAG TCG
dA1	TCCAGCTCTATTACGTCGCCACGGTTTGGCGTGCA
dR1	TGCACGCCAAACCGTGGCGACGTAATAGAGCTGGA
G2_nt	TCTTCCAGGTACACCCTGGCTCTAATACGACTCACTATAGGGAGATTCG TCTCCCCATAGTAATTTGTGCCAGTGCGA
G2_t	TCGCACTGGCACAAATTACTATGGGGAGACGAATCTCCCTATAGTGAG TCG
dA2	TGCGAGGATATTAGAGCCAGGGTGTACCTGGAAGA
dR2	TCTTCCAGGTACACCCTGGCTCTAATATCCTCGCA

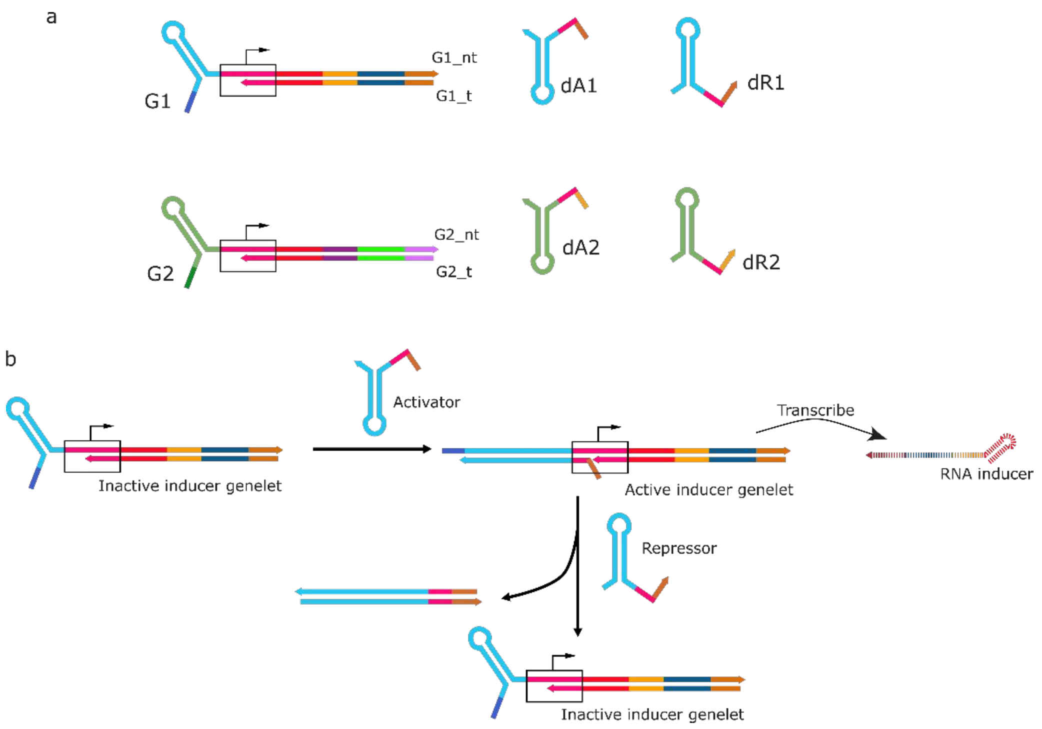

Figure S16. Designed reactions for inducer genelet regulation. (a) DNA species of inducer genelets. (b) Inducer genelet is active when Activator strand bind and complete its promoter domain. The transcribed RNA works as inducer strand to induce switch of assembly state. Transcription is repressed when Repressor strand inhibit the Activator strand.

Table S9. Components for mutually repulsive self-assembly and state switch.

Reagent	Concentration
M^1_1	5 nM
M^1_2	7 nM
M^2_1	5 nM
M^2_2	7 nM
Initial rB1 or rB2	50 nM
G1	5 nM
G2	5 nM
dA1	40 nM
dA2	40 nM
dR1	100 nM
dR2	100 nM
T7 RNAP	2 U/uL
RNase H	0.005 U/uL
YIPP	0.002 U/uL

11. AFM imaging to characterize the structures of the bistable self-assembly system

Besides tracking the tile dimers formation and dissociation kinetics by fluorescence, we also took AFM images to characterize the actual structures of the bistable self-assembly system. Tiles forming Dimer 1 are the same as those in fluorescence measurements. Tiles forming Dimer 2 have the same sticky ends and transcriptional modules as those in fluorescence measurements, but the position of binding edge was changed as shown in the last panel of Figure S1. Thus, Dimer 1 and Dimer 2 could be distinguished by their equal signs layouts: Dimer 1 was identified by the parallel horizontal motif ("= ="), while Dimer 2 identified by the parallel vertical motif ("|| ||") (Figure 5c).

The tile concentration (24 nM) in the bistable self-assembly experiment was initially too high for AFM imaging; thus, it is necessary to dilute the solution by 4- to 5-fold to a final concentration of 5–6 nM just before imaging. While concentration dilution, coupled with temperature change when adding more buffer, can induce dimer dissociation. Besides, distinguishing between monomers and dimers was challenging due to the overlapping, rupturing, and folding of tiles on the mica surface. Consequently, we were unable to reliably calculate the percentage of dimers by counting the absolute number of monomers and dimers. However, it is reasonable to assume that the dilution exerts similar effects on both Dimer 1 and Dimer 2. Thus, by counting the number of each dimer, we can determine which one dominates at different time points. The numbers of each dimer at different time points during state switching experiments in Figure 5b, right panel (*S1* to *S2*), and Figure 6c (*S1* to *S2* to *S1*) were summarized in Table S10 and S11. The resulting dimer distributions confirmed the system's stability and successful state switch. A representative AFM image, taken at 10 hr in the experiment in Figure 6c is provided in Figure S17.

Table S10. Number of each dimer at different time point during state switching from *S1* to *S2* (the procedure in Figure 5b, right panel).

	**1 hr**	**8 hr**
**Dimer 1**	35	1
**Dimer 2**	0	55

Table S11. Number of each dimer at different time point during state switching from *S1* to *S2* and back to *S1* (the procedure in Figure 6c).

	**1 hr**	**4.5 hr**	**10 hr**
**Dimer 1**	48	2	38
**Dimer 2**	0	57	0

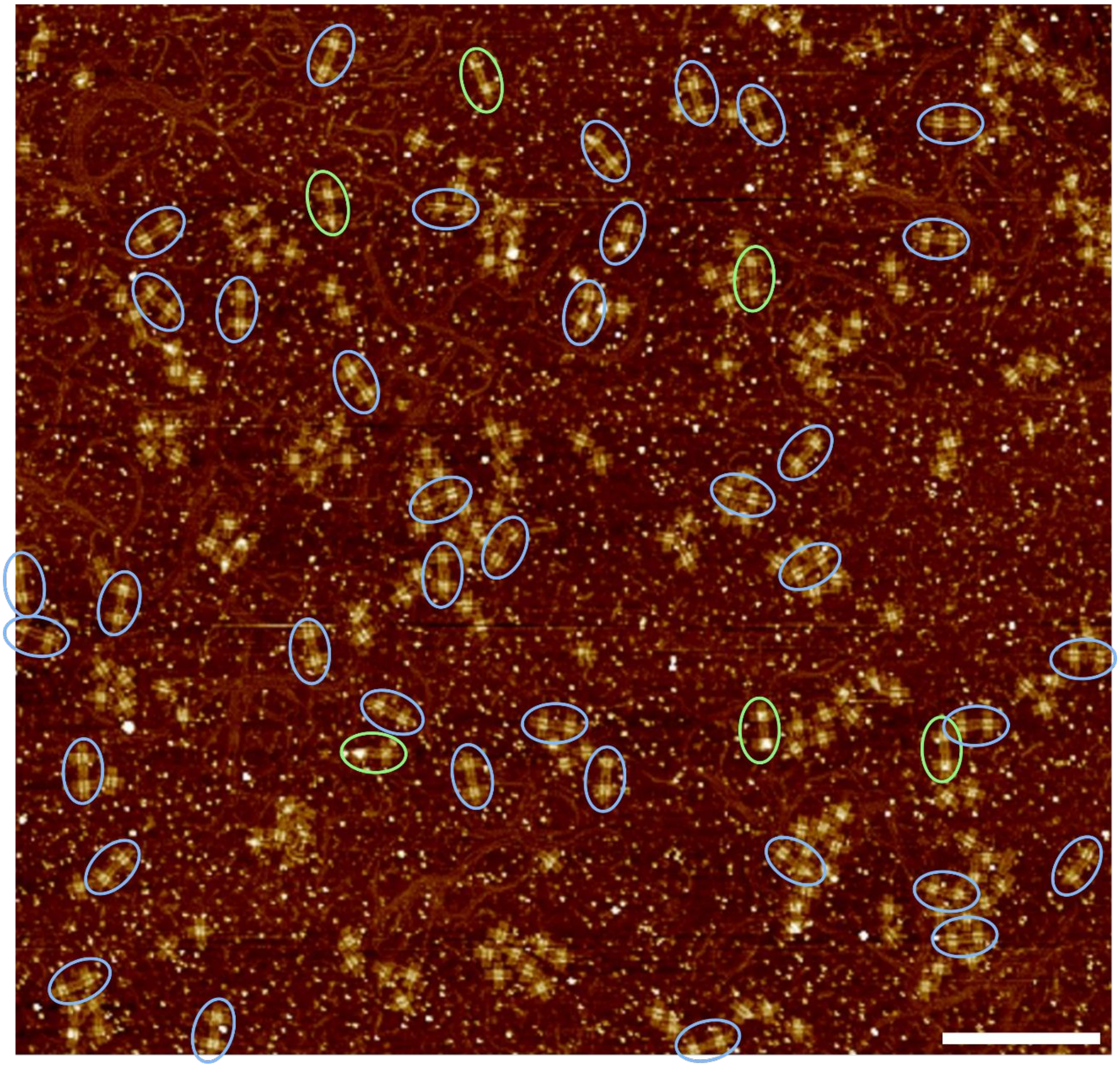

Figure S17. Representative AFM image taken at 10 hr in the experiment in Figure 6c (state switching from *S1* to *S2* and back to *S1)*. Blue ovals indicate Dimer 1 ('= ='). Green ovals indicate dimers that are difficult to identify. Scale bar, 500 nm.

12. Kinetic model

We sought to build a mass action kinetic model of ordinary differential equations (ODEs) that captures the kinetics of the dynamic assembly of the DNA origami tiles. We constructed ODEs describing the kinetics of dimer formation and dissociation, RNA transcription and degradation, and other designed reactions in regulating transcription activity.

12.1 Kinetic model of dimer formation

The binding between sticky ends on two monomers (M_1 and M_2) can produce a dimer (D) (Figure S18a).

$$\frac{d[D]}{dt} = k_{on}[M_1]\,[M_2] - k_{off}[D] \qquad (Eq.8)$$

where k_{on} is the the second order forward reaction rate constant and k_{off} is the first order reverse reaction rate constant. Our experiments have shown that when two monomers are mixed by 5 nM, no dimer was detected by running agarose gel (Figure S5). Thus, the dimerization can be considered as irreversible for tiles in this study. Thus, the reverse reaction term can be ignored.

$$\frac{d[D]}{dt} = k_{on}[M_1]\,[M_2] \qquad (Eq.9)$$

k_{on} was fit to experimental dimerization kinetics (Figure S18b) by numerically integrating the model's differential equations using MATLAB's ode23s function for different parameter sets and minimizing the sum of squared errors between the results predicted by the model and experimental results using MATLAB's lsqnonlin function. The best fit value (2.45×10^{-3} $nM^{-1}\cdot s^{-1}$) was that resulting in the lowest sum of squared errors. All other parameters in this model were determined by the same fitting procedure applied to the corresponding experimental results, unless stated otherwise.

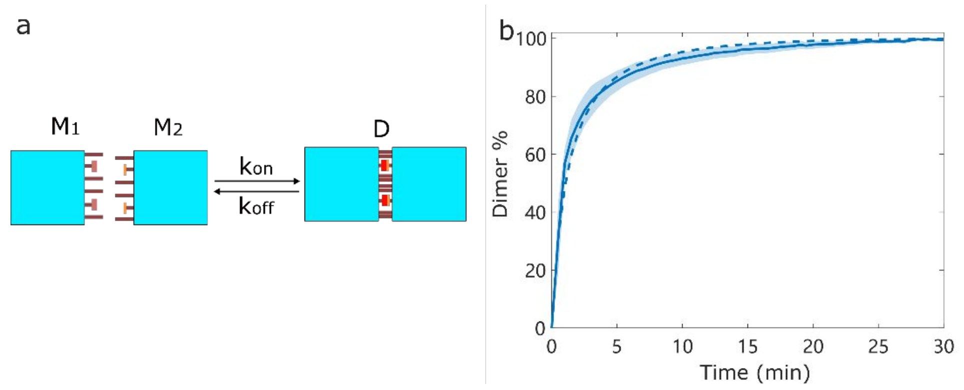

Figure S18. Kinetics of DNA origami tiles dimerization. (a) The binding between sticky ends on two monomers can produce a dimer. (b) The dimerization kinetics was measured by fluorescence. The dimerization kinetics constant (k_{on}) was fit as 2.45×10^{-3} $nM^{-1}\cdot s^{-1}$. Solid line, experiment; dashed line, simulation. Shaded region represents standard variation of 4 measurements.

12.2 The relationship between blocker strands concentration and dimer dissociation

The dimer can be dissociated when the blocker strands block its sticky ends via DNA strands displacement reaction. The dimerization can be considered as a bimolecular reaction, but this is not a solid assumption for the dissociation. There are 4 pairs of sticky ends between M_1 and M_2 whose dissociations are initiated by the same blocker strands. We must focus on the molecular level to study the dissociation process. To simplify, we ignored the interactions between transcriptional modules and activators since the binding energy is much lower than that of sticky ends (6 bps vs. 11 bps).

We first examined how the number of blocked sticky ends affects dimerization at equilibrium. Assuming that blocked sticky ends contribute nothing to dimerization, tiles with *n* sticky ends should exhibit the same dimerization behavior as tiles with *(4–n)* blocked ends. 5 nM M_1 was mixed with 7 nM M_2 with different number of sticky ends. We measured the percentage of M_2 in dimer by running agarose gel and fluorescence measurements and obtained similar results (Table S12).

Table S12. Compare results from agarose gels and fluorescence measurements.

**# of sticky ends**	**4**	**3**	**2**	**1**
**Gel**	101.35%	33.13%	0	0
**Fluorescence**	100%	34.29%	0	0

Based on above results, we built a model describing the dimer dissociation at equilibrium, i.e., the dimer percentage with different DNA blocker (dB) concentrations at equilibrium. We assume that the binding between blocker strands and four sticky ends is independent and identical; thus, the probability of tiles with i blocked sticky ends (P_i) fellows Binomial Distribution:

$$P_i = \binom{4}{i} p^i (1-p)^{4-i} \qquad (Eq.10)$$

p is the probability of blocked sticky ends ($S{:}dB$) that can be calculated as $\frac{[S{:}dB]}{[S]_0}$, where $[S]_0$ is the initial concentration of sticky ends that is 4 (number of sticky ends per tile) times the tile concentration.

We assumed that blocker strands can bind with sticky ends irreversibly; thus,

$$[S{:}dB] = \min\left([dB], [S]_0\right) \qquad (Eq.\ 11)$$

Thus, the probability of dimer can be calculated as the summation of probability of tiles with $(4-i)$ free sticky ends times corresponding Dimer%.

$$P_{dimer} = 1 \times P_0 + 0.3429 \times P_1 + 0 \times P_2 + 0 \times P_3 + 0 \times P_4$$

$$= \binom{0}{4}(1-p)^4 + 0.3429 \times \binom{1}{4}(1-p)^3 p^1$$

$$= (1-p)^4 + 1.3716 \times (1-p)^3 p \qquad (Eq.12)$$

Thus, the concentration of dimer can be calculated as:

$$[D] = [M_1]_0 \times P_{dimer} = [M_1]_0 \times ((1-p)^4 + 1.3716 \times (1-p)^3 p\ )$$

$$= [M_1]_0 \times \left(\left(1 - \frac{[S{:}dB]}{[S]_0}\right)^4 + 1.3716 \times \left(1 - \frac{[S{:}dB]}{[S]_0}\right)^3 \frac{[S{:}dB]}{[S]_0} \right) \qquad (Eq.13)$$

Eq. 13 can be considered as a function of $[S{:}dB]$ to obtain the concentration of dimer ($[D]$), i.e.,

$$[D] = fun\_dimer([S{:}dB\ ]) \qquad (Eq.14)$$

Since we assumed that blocker strands can bind with sticky ends irreversibly, $[S{:}dB]$ can be considered as a function of $[dB]$. Thus, we can predict the concentration of Dimer with addition with different concentrations of DNA blocker strands.

We measured dimer percentages with addition of 0, 1, 2, 5, 10 and 20 nM of blocker strand. To ensure the reaction approached equilibrium, we anneal the mixture from 53°C to 37°C at a rate of 0.7°C/hr. The simulated results from Eq. 14 are in good agreement with the experimental results (Figure 3a).

12.3 Kinetic model of dimer dissociation

We next studied the kinetics of dimer dissociation. The blocker strands can dissociate the complementary sticky ends via DNA strand displacement reaction and inhibit the free sticky ends (Figure S19). Here, S_1 indicates the sticky ends that can bind with blockers.

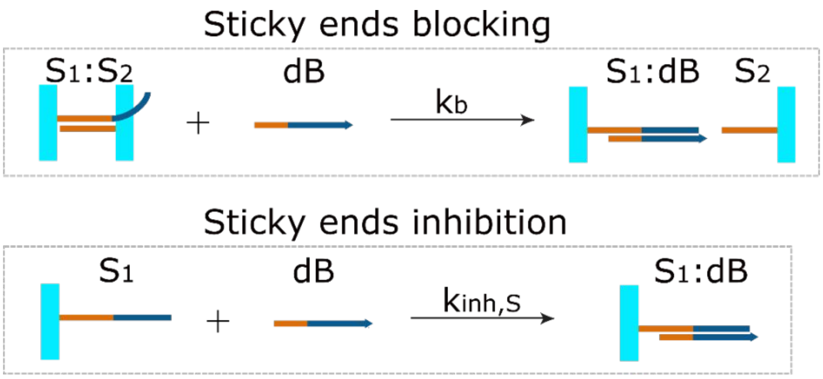

Figure S19. DNA origami tiles dimers can be dissociated by blocker strands.

Here we assume that the kinetics of dimer dissociation can be described as:

$$\frac{d[S_1^i{:}S_2^i]}{dt} = -k_b[S_1^i{:}S_2^i]\,[dB] \qquad (Eq.15)$$

$$\frac{d[S_1^i]}{dt} = -k_{inh}[S_1^i]\,[dB] \qquad (Eq.16)$$

$$\frac{d[S_2^i]}{dt} = k_b[S_1^i{:}S_2^i]\,[dB] \qquad (Eq.17)$$

$$\frac{d[dB]}{dt} = -\sum_{i=1}^{4} k_b\left[S_1^i{:}S_2^i\right][dB] - \sum_{i=1}^{4} k_{inh,S}\left[S_1^i\right][dB] \qquad (Eq.18)$$

$$\frac{d[S_1^i{:}dB]}{dt} = k_b\left[S_1^i{:}S_2^i\right][dB] + k_{inh,S}\left[S_1^i\right][dB] \qquad (Eq.19)$$

We assume that the kinetics for these 4 sticky ends are identical. Thus, Eq. 15-19 can be simplified as:

$$\frac{d[S_1{:}S_2]}{dt} = -k_b[S_1{:}S_2][dB] \qquad (Eq.20)$$

$$\frac{d[S_1]}{dt} = -k_{inh}[S_2][dB] \qquad (Eq.21)$$

$$\frac{d[S_2]}{dt} = k_b[S_1{:}S_2][dB] \qquad (Eq.22)$$

$$\frac{d[dB]}{dt} = -\,4k_b[S_1{:}S_2][dB] - 4k_{inh,S}[S_1][dB] \qquad (Eq.23)$$

$$\frac{d[S_1{:}dB]}{dt} = k_b[S_1{:}S_2][dB] + k_{inh,S}[S_1][dB] \qquad (Eq.24)$$

We can simulate the kinetics of blocked sticky ends ($S_1{:}Blocker$) by Eq. 20-24. We next sought to determine the probability of M_1 in dimer state ($P_{Dimer} = \frac{[D]}{[M_1]_0}$) from the probability of blocked S_1 ($p = \frac{[S_1{:}dB]}{[S_1]_0}$). We have derived a relationship between P_{Dimer} and p at equilibrium (Eq. 14). We assume this relationship still works for self-assembly kinetics. This assumption is valid only when the strand displacement reaction between blocker and complementary sticky ends is much slower than dimer dissociation itself. This means that dimer would dissociate immediately once enough number of sticky ends is blocked. To check if this assumption is reasonable, we compared their reaction times. Previous work has studied kinetics of DNA origami tile dimer dissociation in temperature jump experiments and the measured dissociation constant of DNA origami dimer is ~5×10^{-3} s^{-1}. Thus, the time for 50% dissociation is ~60 s. This time is much shorter than that of blocker-induced dissociation: when adding 20 nM blocker to 5 nM dimer, the time for 50% dissociation is ~1500 s (Figure 3c). In summary, strand displacement limits the reaction rate and we can use Eq. 14 to convert $[S_1{:}Blocker]$ into $[D]$.

We measured the dissociation kinetics by adding 5, 10, 20, 50 nM blocker strands (Figure 3c) and the fitting reaction rate constants (k_b) is 7.43×10^{-6} $nM^{-1}\cdot s^{-1}$. There is no free S1 inhibition during these tests, so the value of $k_{inh,S}$ was determined in subsequent experiments.

12.4 Kinetic model of transcription-regulated dynamic assembly of DNA origami tiles

RNA transcription and degradation are driven by T7 RNAP and RNase H, respectively, and we modeled these reactions using a first order approximation for enzyme kinetics as

previously described for genelet networks. The rate of production of a transcribed RNA is $k_{p,D}[D]$, where $k_{p,D}$ is the apparent production rate constant of transcribed RNA from dimer (D). We assumed that the rates of transcription of monomers were low enough to be neglected. The rate of degradation of RNA is given by $k_d[R{:}S_2]$, where k_d is the apparent degradation rate constant of RNA bound to sticky end ($R{:}S_2$).

We first studied the kinetics of the transcription-induced dissociation of dimers (Figure 3d and Figure S7). We expanded Eq. 20-24 by including RNA blocker transcription and degradation (Figure S20). Once the RNA blocker (rB) bound to S_1 is degraded, the new formed S_1 can bind with S_2 to form dimers. The kinetics of the transcription-induced dissociation of dimers can be characterized by following ODEs.

$$\frac{d[S_1{:}S_2]}{dt} = -k_b[S_1{:}S_2]\,[rB] + k_{on}[S_1]\,[S_2] \qquad (Eq.25)$$

$$\frac{d[S_1]}{dt} = -k_{inh,S}[S_1]\,[rB] - k_{on}[S_1]\,[S_2] + k_d[S_1{:}rB] \qquad (Eq.26)$$

$$\frac{d[S_2]}{dt} = k_b[S_1{:}S_2]\,[rB] - k_{on}[S_1]\,[S_2] \qquad (Eq.27)$$

$$\frac{d[rB]}{dt} = k_{p,G}[G|rB] - 4k_b[S_1{:}S_2]\,[rB] - 4k_{inh,S}[S_1]\,[rB] \qquad (Eq.28)$$

$$\frac{d[S_1{:}rB]}{dt} = 4k_b[S_1{:}S_2]\,[rB] + 4k_{inh,S}[S_1]\,[rB] - k_d[S_1{:}rB] \qquad (Eq.29)$$

$$[D] = fun\_dimer([S_1{:}rB\,]) \qquad (Eq.30)$$

The values of k_{on} and k_b were using the previous fit results. The values of $k_{p,G}$ and k_d were obtained by fitting experimental results in Figure 3d as 0.0197 s^{-1}, 1.10×10^{-4} s^{-1}.

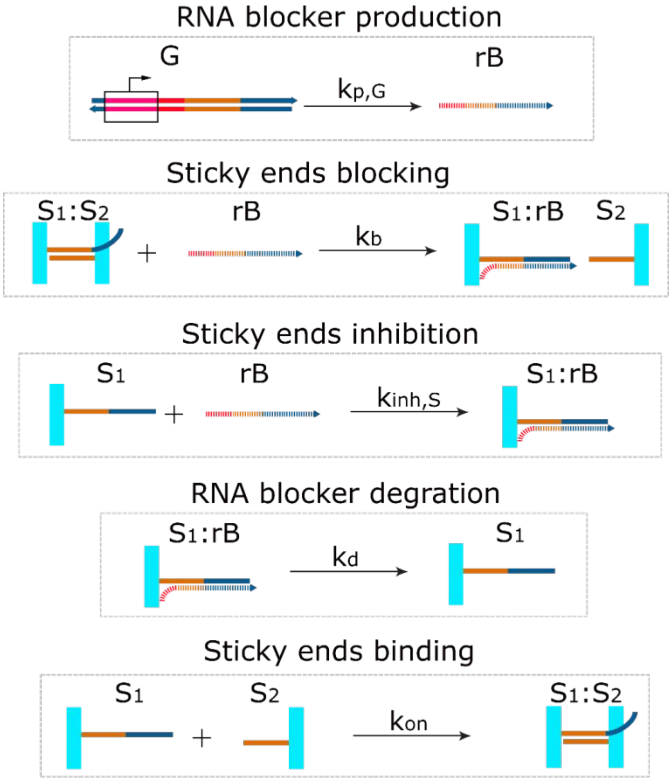

Figure S20. Reactions included in transcription-induced dimer dissociation.

12.5 Kinetic model of mutual repulsive DNA origami tiles assembly system

We next sought to construct the kinetic model of bistable dimerization system consisting of two tile pairs whose transcripts serve as blocker strands (rB^1 and rB^2) to another. State can be switched by inducer strands (rI^1 and rI^2) that can remove blocker strands from sticky ends and inhibit free blocker strands. Reactions in this system were enumerated in Figure S21. Superscript 1 and 2 indicate these two pairs of tiles and corresponding blocker and inducer strands. The kinetics of the mutual repulsive DNA origami tiles assembly system can be characterized by following ODEs.

$$\frac{d[S_1^1{:}S_2^1]}{dt} = -k_b[S_1^1{:}S_2^1]\,[rB^2] + k_{on}[S_1^1]\,[S_2^1] \qquad (Eq.31)$$

$$\frac{d[S_1^2{:}S_2^2]}{dt} = -k_b[S_1^2{:}S_2^2]\,[rB^1] + k_{on}[S_1^2]\,[S_2^2] \qquad (Eq.32)$$

$$\frac{d[S_1^1]}{dt} = -k_{inh,s}[S_1^1]\,[rB^2] - k_{on}[S_1^1]\,[S_2^1] + k_d[S_1^1{:}rB^2] \qquad (Eq.33)$$

$$\frac{d[S_1^2]}{dt} = -k_{inh,s}[S_1^2]\,[rB^1] - k_{on}[S_1^2]\,[S_2^2] + k_d[S_1^2{:}rB^1] \qquad (Eq.34)$$

$$\frac{d[S_2^1]}{dt} = k_b[S_1^1{:}S_2^1]\,[rB^2] - k_{on}[S_1^1]\,[S_2^1] \qquad (Eq.35)$$

$$\frac{d[S_2^2]}{dt} = k_b[S_1^2{:}S_2^2]\,[rB^1] - k_{on}[S_1^2]\,[S_2^2] \qquad (Eq.36)$$

$$\frac{d[rB^1]}{dt} = 2k_{p,D}[D^1] - 4k_b\left[S_1^2{:}S_2^2\right][rB^1] - 4k_{inh,S}\left[S_1^2\right][rB^1] - k_{inh,R}[rI^1][rB^1] \qquad (Eq.37)$$

$$\frac{d[rB^2]}{dt} = 2k_{p,D}[D^2] - 4k_b\left[S_1^1{:}S_2^1\right][rB^2] - 4k_{inh,S}\left[S_1^1\right][rB^2] - k_{inh,R}[rI^2][rB^2] \qquad (Eq.38)$$

$$\frac{d[S_1^1{:}rB^2]}{dt} = k_b\left[S_1^1{:}S_2^1\right][rB^2] + k_{inh,S}\left[S_1^1\right][rB^2] - k_d[S_1^1{:}rB^2] - k_{dis}[rI^2]\left[S_1^1{:}rB^2\right] \qquad (Eq.39)$$

$$\frac{d[S_1^2{:}rB^1]}{dt} = k_b\left[S_1^2{:}S_2^2\right][rB^1] + k_{inh,S}\left[S_1^2\right][rB^1] - k_d[S_1^2{:}rB^1] - k_{dis}[rI^1]\left[S_1^2{:}rB^1\right] \qquad (Eq.40)$$

$$\frac{d[r_{Inducer}^1]}{dt} = -k_{inh,B}[rI^1][rB^1] - k_{dis}[rI^1]\left[S_1^2{:}rB^1\right] \qquad (Eq.41)$$

$$\frac{d[r_{Inducer}^2]}{dt} = -k_{inh,B}[rI^2][rB^2] - k_{dis}[rI^2]\left[S_1^1{:}rB^2\right] \qquad (Eq.42)$$

$$[D^1] = fun\_dimer([S_1^1{:}rB^1]) \qquad (Eq.43)$$

$$[D^2] = fun\_dimer([S_1^2{:}rB^2]) \qquad (Eq.44)$$

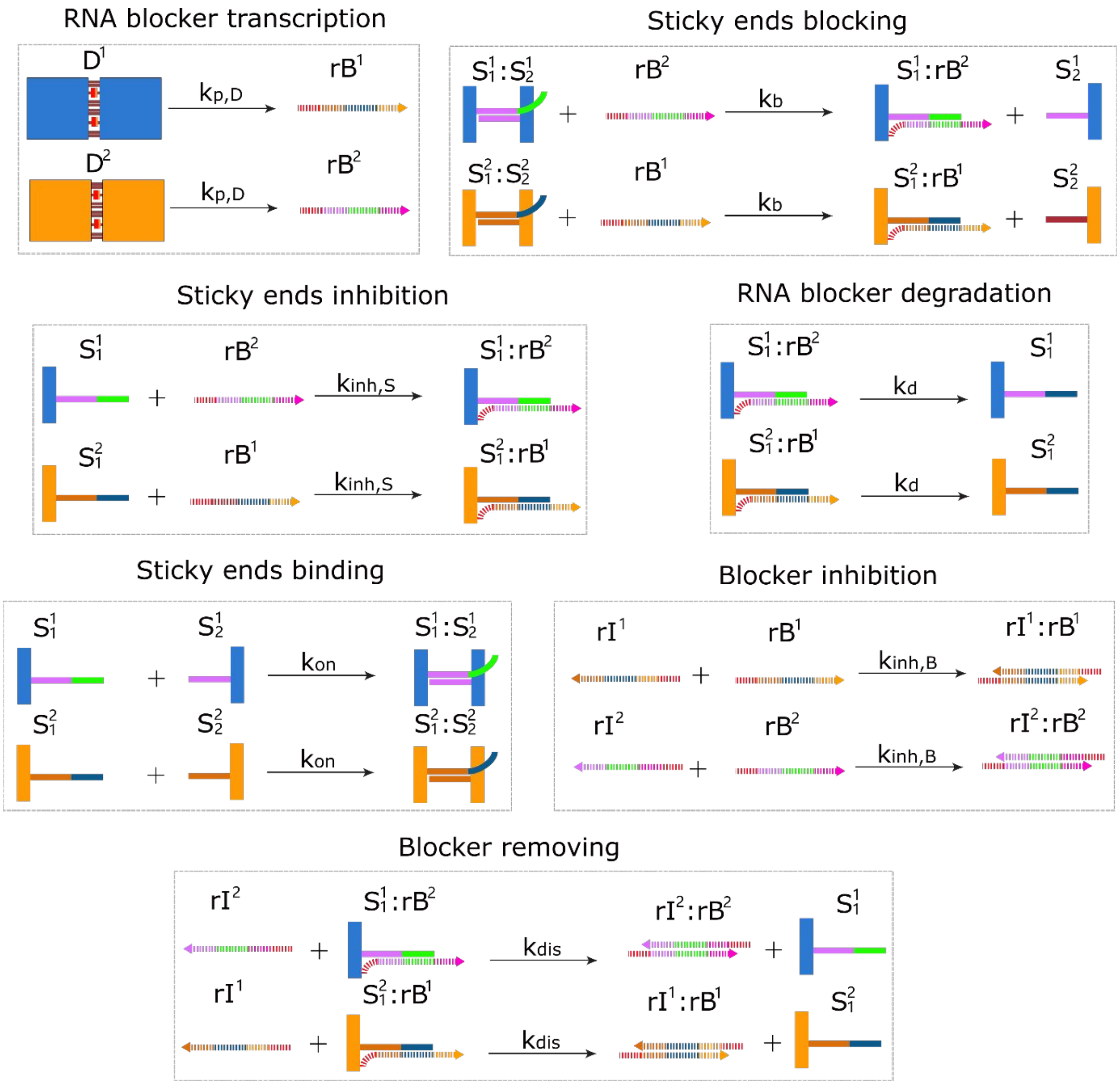

Figure S21. Reactions included in mutual repulsive DNA origami tiles assembly system.

To better understand the influence of inducer concentrations on switching dynamics, we simulated the system's responses to different levels of rI1 and compared them with experimental data (Figure S22a, d). Specifically, we examined the simulated concentrations of the blocker (rB1) and blocked sticky ends ($S_1^2{:}rB^1$) following the addition of 300 nM (Figure S22b, c) and 100 nM (Figure S22e, f) inducer strands. The initial state was State 1 (Dimer 1 takes dominance and Dimer 2 is dissociated) set by adding 50 nM rI1 to a mixture of 5 nM Dimer 1 and 5 nM Dimer 2. ~20 nM rI1 was bound to the four sticky ends of Dimer 2. Consequently, the free rI1 concentration was ~30 nM (Figure S22b, e), and nearly all sticky ends on Dimer 2 were blocked (Figure S22c, f). Upon the addition of T7 RNAP, Dimer 1 produced rB1, maintaining Dimer 2 in a dissociated state. After 2 hours of incubation, rB1 accumulated to ~150 nM. Experimentally, 300 nM rI1 was sufficient to switch the system's state, whereas 100 nM rI1 was not, consistent with the simulation results. 300 nM rI1 is enough to inactivates any excess rB1 (Figure S22b) and reactivate blocked sticky ends on Dimer 2 immediately (Figure S22c). The excessive rI1 can further repress the

expression of Dimer 1 by inhibiting the newly form rB1. Such an inhibition allowed Dimer 2 to reform, leading to the transcription of rB2 and the dissociation of Dimer 1. By contrast, 100 nM rI1 was insufficient to inhibit all free rB1 (Figure S22e), resulting in only minimal removal of rB1 bound to Dimer 2 sticky ends (Figure S22f). Consequently, Dimer 1 continued to produce rB1, preventing Dimer 2 reformation and leaving the system in its initial state.

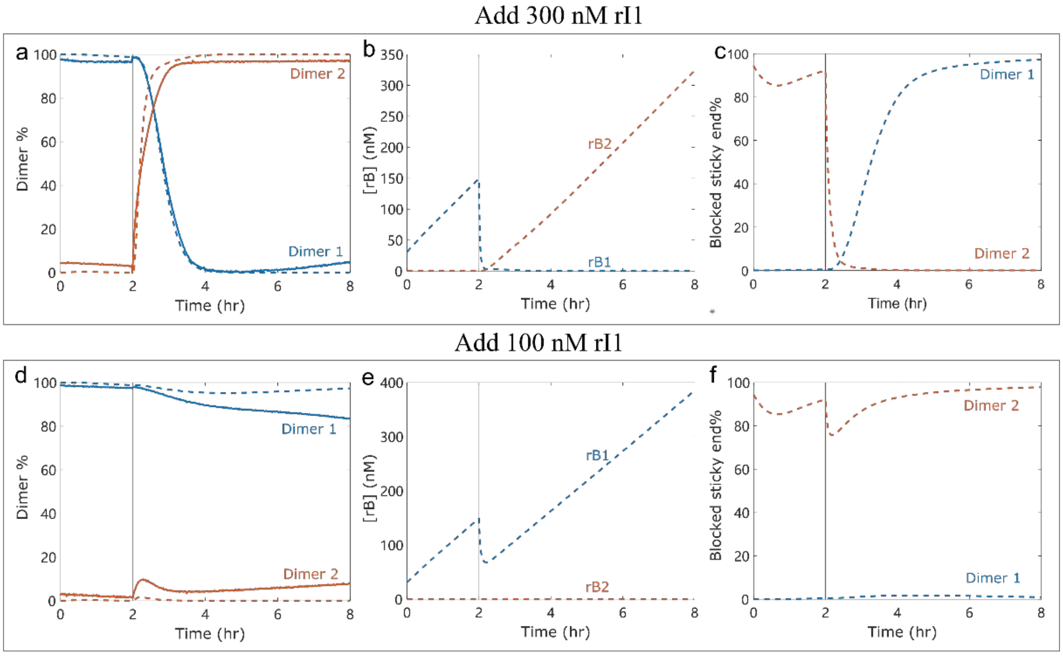

Figure S22. Comparison of the simulated dynamics of dimer, RNA blocker and blocked sticky end of adding 300 nM (a-c) and 100 nM rI1 (e-f) after 2 hr incubation for state switch. Solid lines in a, d indicate experimental measured Dimer%. Solid lines, experimental Dimer%; dashed lines, simulations.

RNA inducers (rI^1 and rI^1) can also be transcribed by other genelets ($G_1|rI^1$ and $G_2|rI^2$). Their T7 RNAP promoter sequence is not double-stranded, so little transcription occurs. When the activator strands (A^1 and A^2) completes the promoter sequence, high transcription occurs from a genelet:activator complex ($A^1{:}G^1|rI^1$ and $A^2{:}G^2|rI^2$). The active genelet can be turned off again when repressor strands (R^1 and R^2) remove the activator strands (Figure S23).

The kinetics of activation and repression of $G_1|rI^1$ and $G_2|rI^2$ can be described by Eq. 45-54 that can be included in the model of mutual repulsive DNA origami tiles assembly system.

$$\frac{d[rI^1]}{dt} = k_{p,G_1} A^1{:}G^1|rI^1 - k_{inh}[rI^1]\,[rB^1] - k_{dis}[rI^1]\,[S_1^2{:}rB^1] \qquad (Eq.45)$$

$$\frac{d[rI^2]}{dt} = k_{p,G_2} A^2{:}G^2|rI^2 - k_{inh}[rI^2]\,[rB^2] - k_{dis}[rI^2]\,[S_1^1{:}rB^2] \qquad (Eq.46)$$

$$\frac{d[A^1{:}G^1\,|\,rI^1]}{dt} = k_{act}[A^1]\,[G^1\,|\,rI^1] - k_{rep}[R^1]\,[A^1{:}G^1\,|\,rI^1] \qquad (Eq.47)$$

$$\frac{d[A^2{:}G^2|rI^2]}{dt} = k_{act}[A^2]\left[G^2\,\middle|\,rI^2\right] - k_{rep}[R^2]\,[A^2{:}G^2|rI^2] \qquad (Eq.48)$$

$$\frac{d[A^1]}{dt} = -k_{act}[A^1]\left[G^1\,\middle|\,rI^1\right] - k_{inh,A}[R^1][A^1] \qquad (Eq.49)$$

$$\frac{d[A^2]}{dt} = -k_{act}[A^2]\left[G^2\,\middle|\,rI^2\right] - k_{inh,A}[R^2][A^2] \qquad (Eq.50)$$

$$\frac{d\left[G^1\,\middle|\,rI^1\right]}{dt} = -k_{act}[A^1]\left[G^1\,\middle|\,rI^1\right] + k_{rep}[R^1]\left[A^1{:}G^1\,\middle|\,rI^1\right] \qquad (Eq.51)$$

$$\frac{d\left[G^2\,\middle|\,rI^2\right]}{dt} = -k_{act}[A^2]\left[G^2\,\middle|\,rI^2\right] + k_{rep}[R^2]\,[A^2{:}G^2|rI^2] \qquad (Eq.52)$$

$$\frac{d[R^1]}{dt} = -k_{rep}[R^1]\left[A^1{:}G^1\,\middle|\,rI^1\right] - k_{inh,A}[R^1][A^1] \qquad (Eq.53)$$

$$\frac{d[R^2]}{dt} = -k_{rep}[R^2]\,[A^2{:}G^2|rI^2] - k_{inh,A}[R^2][A^2] \qquad (Eq.54)$$

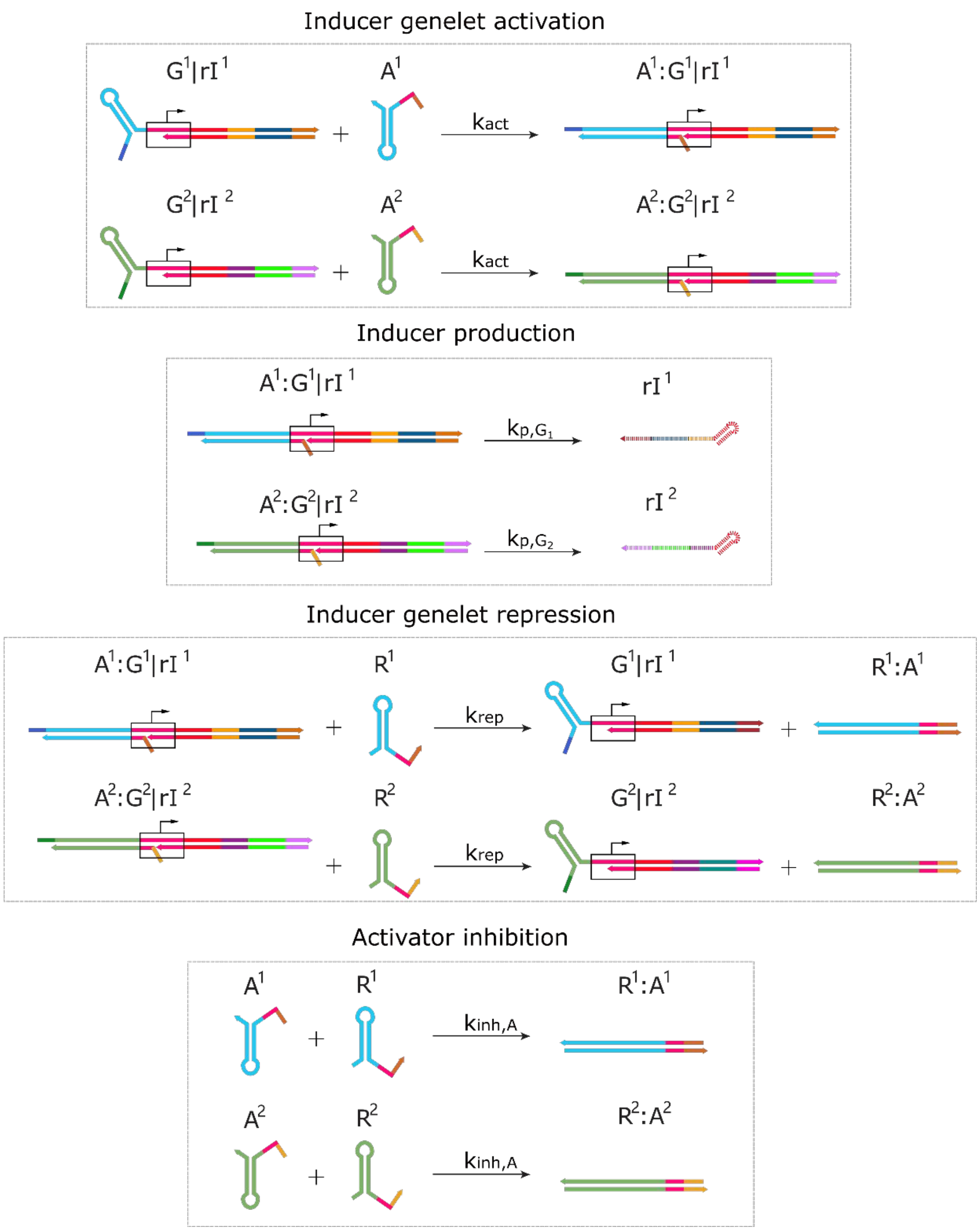

Figure S23. Reactions included in Inducer genelet activation and repression.

Reaction constants related to genelet activation and repression used in simulation in Figure 6a-c were summarized in Table S13. They are in the same order of magnitude as results in previous work[6].

Table S13. Reaction constants in simulation.

k_{on}	2.45×10^{-3} nM$^{-1}\cdot$s^{-1}
k_b	7.43×10^{-6} nM$^{-1}\cdot$s^{-1}
$k_{p,G}$	0.0197 s^{-1}

k_d	1.10×10^{-4} s^{-1}
$k_{p,D}$	1.8×10^{-3} s^{-1}
$k_{inh,S}$	4.8×10^{-5} nM^{-1}·s^{-1}
$k_{inh,B}$	4.8×10^{-5} nM^{-1}·s^{-1}
k_{dis}	1.6×10^{-5} nM^{-1}·s^{-1}
k_{p,G_1}	0.016 s^{-1}
k_{p,G_2}	0.011 s^{-1}
k_{act}	2.2×10^{-5} nM^{-1}·s^{-1}
k_{rep}	2.6×10^{-5} nM^{-1}·s^{-1}
$k_{inh,A}$	5.0×10^{-5} nM^{-1}·s^{-1}

12.6 Simulation of other dynamic systems

The modules in this work have the potential to be combined to achieve other dynamics, such as oscillation[8, 9] and generation of pulse[6, 10]. Here we used the kinetic model in this work to demonstrate how these dynamics can be constructed by combining different DNA origami tile pairs.

One common network architecture is "repressilator", which has odd number of nodes repress one another in a circular pattern[9]. The dynamic DNA origami tiles assembly system in this work has the potential to achieve oscillations by choosing odd numbers of tile pair whose transcript serves as blocker to dissociate another in a circular pattern. We simulated repressilators with three and five tile pairs. For all both systems we were able to identify conditions that gave rise to persistent oscillations (Figure S24a, b). The ODEs involved were shown in Eq. 55-60. Dimer D^i produces RNA rB^i that dissociates Dimer D^k and it can be dissociated by RNA rB^j produced by Dimer D^j.

$$\frac{d[S_1^i{:}S_2^i]}{dt} = -k_b[S_1^i{:}S_2^i]\,[rB^j] + k_{on}[S_1^i]\,[S_2^i] \qquad (Eq.55)$$

$$\frac{d[S_1^i]}{dt} = -k_{inh,S}[S_1^i]\,[rB^j] - k_{on}[S_1^i]\,[S_2^i] + k_d[S_1^i{:}rB^j] \qquad (Eq.56)$$

$$\frac{d[S_2^i]}{dt} = k_b[S_1^i{:}S_2^i]\,[rB^j] - k_{on}[S_1^i]\,[S_2^i] \qquad (Eq.57)$$

$$\frac{d[rB^i]}{dt} = k_{p,G}[D^i] - 4k_b[S_1^k{:}S_2^k]\,[rB^i] - 4k_{inh,S}[S_1^k]\,[rB^i] \qquad (Eq.58)$$

$$\frac{d[S_1^i{:}rB^j]}{dt} = k_b[S_1^i{:}S_2^i]\,[rB^j] + k_{inh,S}[S_1^i]\,[rB^j] - k_d[S_1^i{:}rB^j] \qquad (Eq.59)$$

$$[D^i] = fun\_dimer([S_1^i{:}rB^j]) \qquad (Eq.60)$$

Same set of tile pairs can show other dynamics by changing the interaction between them. We also studied the generation of pulse, i.e., two tiles form a dimer and dissociate in a programmable mode (Figure S24c). This system is initiated by adding RNA blocker rB^2 and rB^5; thus, the dimer D^3 and D^4 are dissociated. When transcription is turned on, dimer D^1 produces RNA blocker rB^1 that dissociates the dimer D^2 and D^5. Thus, the production of RNA blocker rB^2 and rB^5 is repressed, causing the formation of dimer D^3 and D^4. The formation of D^3 will further produce RNA blocker rB^3 that dissociates dimer D^4. We simulated this system and the magnitude and last time of dimer D^4 pulse can be tuned by changing the concentrations of dimer D^2 (4, 8, 12 nM).

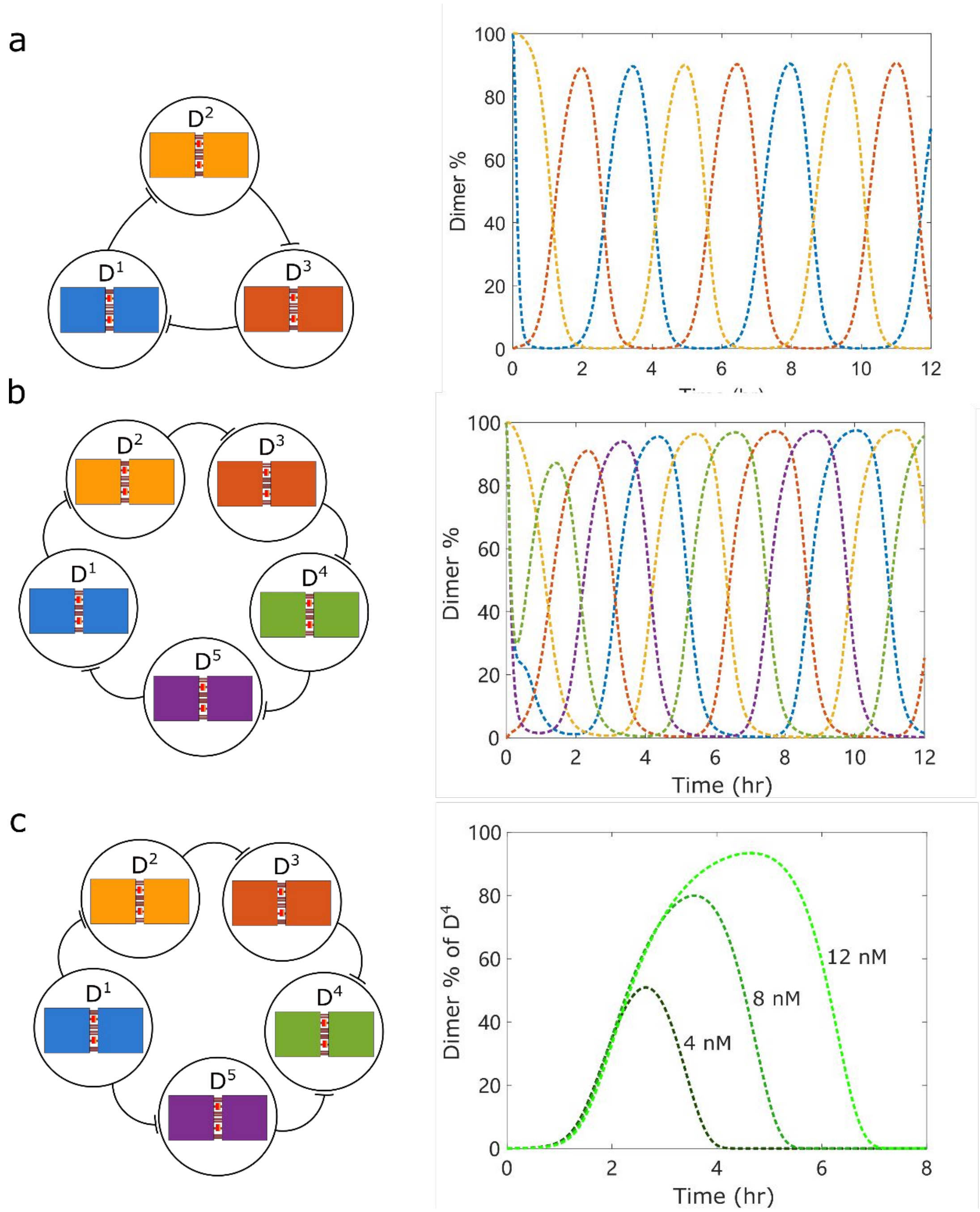

Figure S24. Simulation of other dynamics. (a) 3 tile pairs repressilator simulation. (b) 5 tile pairs repressilator simulation. In all simulations, all tile pairs' concentrations are 5 nM, k_b = 2×10^{-4} $nM^{-1}\cdot s^{-1}$, $k_{inh,S}$ = 1×10^{-3} $nM^{-1}\cdot s^{-1}$, k_d = 2.2×10^{-3} s^{-1}. (c) Network diagram for pulse generation. The magnitude and last time of dimer D^4 pulse can be tuned by changing the concentrations of dimer D^2. In all simulations, all tile pairs' concentrations are 5 nM except dimer D^2, k_b = 2×10^{-4} $nM^{-1}\cdot s^{-1}$, $k_{inh,S}$ = 1×10^{-3} $nM^{-1}\cdot s^{-1}$, k_d = 3.2×10^{-4} s^{-1}.

REFERENCES

(1) Liu, W.; Zhong, H.; Wang, R.; Seeman, N. C. Crystalline two-dimensional DNA origami arrays. *Angewandte Chemie (International ed. in English)* **2011**, *50* (1), 264.

(2) Martin, C. T.; Coleman, J. E. Kinetic-Analysis of T7 Rna-Polymerase Promoter Interactions with Small Synthetic Promoters. *Biochemistry-Us* **1987**, *26* (10), 2690-2696. DOI: DOI 10.1021/bi00384a006.

(3) Schaffter, S. W.; Green, L. N.; Schneider, J.; Subramanian, H. K.; Schulman, R.; Franco, E. T7 RNA polymerase non-specifically transcribes and induces disassembly of DNA nanostructures. *Nucleic acids research* **2018**, *46* (10), 5332-5343.

(4) Kalra, S.; Donnelly, A.; Singh, N.; Matthews, D.; Del Villar-Guerra, R.; Bemmer, V.; Dominguez, C.; Allcock, N.; Cherny, D.; Revyakin, A. Functionalizing DNA Origami by Triplex-Directed Site-Specific Photo-Cross-Linking. *Journal of the American Chemical Society* **2024**, *146* (19), 13617-13628.

(5) Schaffter, S. W.; Schulman, R. Building in vitro transcriptional regulatory networks by successively integrating multiple functional circuit modules. *Nature chemistry* **2019**, *11* (9), 829-838.

(6) Schaffter, S. W.; Chen, K.-L.; O'Brien, J.; Noble, M.; Murugan, A.; Schulman, R. Standardized excitable elements for scalable engineering of far-from-equilibrium chemical networks. *Nature chemistry* **2022**, *14* (11), 1224-1232.

(7) Sobek, J.; Schlapbach, R. Dependence of fluorescence quenching of CY3 oligonucleotide conjugates on the oxidation potential of the stacking base pair. *Molecules* **2020**, *25* (22), 5369.

(8) Kim, J.; Winfree, E. Synthetic in vitro transcriptional oscillators. *Molecular systems biology* **2011**, *7* (1), 465.

(9) Niederholtmeyer, H.; Sun, Z. Z.; Hori, Y.; Yeung, E.; Verpoorte, A.; Murray, R. M.; Maerkl, S. J. Rapid cell-free forward engineering of novel genetic ring oscillators. *elife* **2015**, *4*, e09771.

(10) Mangan, S.; Alon, U. Structure and function of the feed-forward loop network motif. *Proceedings of the National Academy of Sciences* **2003**, *100* (21), 11980-11985.
